# Poly(pyridinium salt)s Containing 9,9-Bis(4-aminophenyl)fluorene Moieties with Various Organic Counterions Exhibiting Both Lyotropic Liquid-Crystalline and Light-Emitting Properties

**DOI:** 10.3390/polym17131785

**Published:** 2025-06-27

**Authors:** Pradip K. Bhowmik, David King, Haesook Han, András F. Wacha, Matti Knaapila

**Affiliations:** 1 Department of Chemistry and Biochemistry, University of Nevada Las Vegas, 4505 Maryland Parkway, Box 454003, Las Vegas, NV 89154, USA; david.king@unlv.edu (D.K.); hanh3@unlv.nevada.edu (H.H.); 2 Research Center for Natural Sciences, Institute of Materials and Environmental Chemistry, Magyar Tedesko Köútja 2, H-1117 Budapest, Hungary; wacha.andras@ttk.hu; 3 Department of Physics, Norwegian University of Science and Technology, Høgskoleringen 5, 7491 Trondheim, Norway; matti.knaapila@ntnu.no

**Keywords:** poly(pyridinium salt)s, metathesis reaction, lyotropic liquid-crystalline phase, conjugated polyelectrolytes (CPEs), hairy-rod supramolecules, polarizing optical microscopy, small angle X-ray scattering (SAXS), luminescence, UV-Vis spectroscopy

## Abstract

Main-chain conjugated and non-conjugated polyelectrolytes are an important class of materials that have many technological applications ranging from fire-retardant materials to carbon-nanotube composites, nonlinear optical materials, electrochromic materials for smart windows, and optical sensors for biomolecules. Here, we describe a series of poly(pyridinium salt)s-fluorene containing 9,9-bis(4-aminophenyl)fluorene moieties with various organic counterions that were synthesized using ring-transmutation polymerization and metathesis reactions, which are non-conjugated polyelectrolytes. Their chemical structures were characterized by Fourier transform infrared (FTIR), proton (^1^H) and fluorine 19 (^19^F) nuclear magnetic resonance (NMR) spectrometers, and elemental analysis. They exhibited polyelectrolytic behavior in dimethyl sulfoxide. Their lyotropic liquid-crystalline phases were examined by polarizing optical microscopy (POM) and small angle X-ray scattering (SAXS) studies. Their emission spectra exhibited a positive solvatochromism on changing the polarity of solvents. They emitted greenish-yellow lights in polar organic solvents. They formed aggregates in polar aprotic and protic solvents with the addition of water (*v*/*v*, 0–90%), whose λ_em_ peaks were blue shifted.

## 1. Introduction

Phenylated poly(pyridinium salt)s are a class of main-chain cationic polymers known as ionenes. They are usually prepared by the ring-transmutation polymerization reaction of bispyrylium salts and diamines and metathesis reactions. Depending on the chemical structures of bispyrylium salts and diamines, they can be π-conjugated or non-conjugated ionenes. Various non-conjugated ionenes exhibit thermotropic liquid-crystalline (LC) and light-emitting properties in both solution and solid state. Additionally, π-conjugated and even non-conjugated ionic polymers exhibit lyotropic LC phases in both protic and aprotic solvents and light-emitting properties in both solution and solid state depending on their chemical microstructures [1,2,3,4]. Many of their properties are akin to LC hairy-rod polymers and supramolecular hairy-rod polymers [5,6,7,8]. They are also called functional polymers, since they can be used for the synthesis of carbon-nanotube composites [9], fire-retardant polymers [10], electrochromic materials for smart windows [11], and nonlinear optical properties [12]. Additionally, they are used for the detection of various types of biomolecules based on the optical properties of phenylated pyridinium and aromatic diamine moieties. Notable examples include the detection of Pseudomonas fluorescens DNA [13], homogeneous DNA [14], calf thymus DNA [15,16,17], and heparin [18]. They belong to a class of main-chain conjugated (MCPs) and non-conjugated polyelectrolytes wherein charges are located along the backbone of the polymer chains. Recently, two interesting review articles appeared in the literature [19,20] on the different methods of their synthesis and applications.

On the other hand, charged groups are attached to the side chains of the conjugated (SCPs), and non-conjugated polymer chains are known as side chain polyelectrolytes. Notable examples of SCPs include poly(thiophene)s, poly(fluorene)s, poly(p-phenylene)s, poly(p-phenylenevinylene)s, poly(p-phenylene ethynylene)s, and poly(diketopyrrolopyrrole)s, among others. Generally, charged groups including quaternary ammonium, phosphonium, carboxyl, sulfonic, phosphate, pyridinium, and imidazolium groups were incorporated in SCPs via post-polymerization reactions that pose challenging tasks [21,22]. Nonetheless, these SCPS are an important class of polyelectrolytes that find versatile applications in modern science and technology including optical sensors for molecular and biomolecular targets, organic electronic applications, and biological applications such as cellular imaging and photodynamic therapy. The structural and photophysical templating of SCPs with ss-DNA was also reported [23]. Additionally, it is interesting to note that the cationic SCP/DNA polyplexes are used in nucleic acid delivery [24], cationic phosphonium SCPs are used for antibacterial applications [25], and even the SCPs are used as a defect-passivating hole injection layer for efficient and stable perovskites light-emitting diodes [26].

**Scheme 1:**
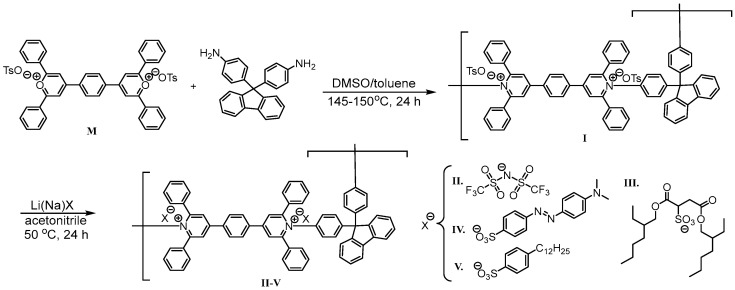
Synthesis of poly(pyridinium salt)s-9,9-bis(4-aminophenyl)fluorene moieties, **I**–**V**.

In this article, we describe the synthesis of a series of poly(pyridinium salt)s containing bulky fluorene moieties in the polymer backbone as well as organic counterions including as tosylate, triflimide, docusate, methyl orange, and dodecyl benzene sulfonate using ring-transmutation polymerization [1,5] as well as metathesis reactions and the characterization of their lyotropic liquid-crystalline (LC) properties in polar organic solvents. The general structure and designations of these ionic polymers, **I**–**V**, which were prepared and characterized for this study, are shown in Scheme 1. They are novel examples of poly(pyridinium salt)s with fluorene moieties in the main chain that exhibit lyotropic LC properties in various organic solvents. They also exhibit photoluminescence both in solution and in the solid state. Therefore, the synthesis and characterization of both lyotropic liquid-crystalline and light-emitting properties of poly(pyridinium salt)s with fluorene containing moieties by using several experimental techniques are of significant interest for the development of main-chain cationic polyelectrolytes. The techniques used for the characterization of these main-chain ionic polymers include Fourier transformation infrared (FTIR) spectroscopy, Fourier transform nuclear magnetic resonance (FT-NMR) spectroscopy, elemental analysis, solution viscosity, dynamic light scattering (DLS), polarizing optical microscopy (POM), and small angle X-ray scattering (SAXS) studies. Their photoluminescence properties both in solutions of polar solvents and in the solid state using luminescence spectrometer are also included.

## 2. Materials and Methods

### 2.1. Instrumentation

The FTIR spectra were recorded with a Shimadzu infrared spectrometer (Shimadzu Scientific Instruments, Inc., Moorpark, CA, USA). Polymers **I**–**V** were prepared by coating NaCl plates with various polymers and subsequently vacuum dried at 70 °C overnight. The ^1^H and ^19^F and spectra were obtained using VNMR 400 spectrometer (Varian Inc., Palo Alto, CA, USA) operating at 400 MHz for ^1^H, 100 MHz for ^13^C, and 376 MHz for ^19^F nuclei with three RF channels at room temperature, and chemical shifts were referenced to tetra methylsilane (TMS) for proton nuclei and trichlorofluoromethane (CFCl_3_) for fluorine nuclei. The NMR samples were prepared by applying gentle heating to dissolve the polymer in *d*_6_-DMSO. Elemental analysis was performed by Atlantic Microlab Inc. (Norcross, GA, USA). Molecular weight of polymer **I** was estimated in terms of dynamic light scattering (DLS) using Marvern Zetasizer Nano. Several samples with their concentrations ranging from 0.25 to 5 mg/mL dissolved in DSMO were measured at room temperature with and without shape correction using the Rayleigh Equation and Debye plots. The second virial coefficient was found to be larger than 0 and solvent thus deemed good.

Lyotropic liquid crystal (LC) phases of the polymers were examined using a polarized optical microscope (POM, Nikon, Tokyo, Japan, Model Labophot 2) equipped with crossed polarizers. Samples of polymers for lyotropic LC properties were made by dissolving known amounts of polymer into known amounts of organic solvents (DMSO, CH_3_CN, or MeOH). The UV–Vis absorption spectra of polymer solutions in spectrograde organic solvents were recorded at room temperature using Varian Cary 50 Bio UV–Visible spectrophotometer (Agilents Technologies, Santa Clara, CA, USA) in quartz cuvettes. Photoluminescence spectra in solutions and thin films were recorded with a Perkin Elmer LS 55 luminescence spectrometer (Perkin Elmer, Akron, OH, USA) with a xenon lamp light source. Absolute quantum yields of polymers **I**–**V** in both methanol solution and powdered form were measured with a Horiba Fluorolog fluorimeter (HORIBA Instruments Inc., Irvine, CA, USA) equipped with an integrating sphere.

### 2.2. Materials

Lithium triflimide, methyl orange, dodecylbenzene sulfonic acid (sodium salt), dioctyl sulfosuccinate (sodium salt), and common organic solvents were purchased from Sigma-Aldrich (Milwaukee, WI, USA) and TCI America (Portland, OR, USA) and used without further purification. For synthesis and purification purposes, reagent grade solvents including acetonitrile, ethanol, methanol, ethyl acetate, spectral grade dimethyl sulfoxide (DMSO), acetonitrile, methanol, chloroform, and tetrahydrofuran were used as obtained from Sigma-Aldrich. The spectrograde organic solvents were also obtained from Sigma-Aldrich. Deuterated solvents were obtained from Cambridge Isotope Laboratories, Inc. (Tewksbury, MA, USA).

### 2.3. Monomers Synthesis

The 4,4′-(1,4-phenylene)bis(2,6-diphenylpyrylium)ditosylate, **M**, was synthesized according to the reported procedure [1]. The 9,9-bis(4-aminophenyl)fluorene was purchased from Tokyo Kasei Kogyo Co., Ltd., Tokyo, Japan, and purified by recrystallization from chloroform/hexane. It showed a melting endotherm (*T_m_*) at peak maximum of 237 °C in the DSC thermogram obtained at a heating rate of 10 °C/min. Its reported mp = 236–237 °C [27]. In the subsequent cooling cycle at identical rate, the absence of the crystallization exotherm suggested it formed the glassy state from the melt. In the second heating cycle, it showed a glass transition temperature (*T_g_*), a cold crystallization exotherm, and a *T_m_* at slightly reduced temperature with a peak maximum of 230 °C.

### 2.4. Synthesis of Polymer ***I***

The synthesis of polymer **I** was performed according to the procedure reported in the literature [1] by reacting 4,4′-(1,4-phenylene)bis(2,6-diphenylpyrylium)ditosylate, **M**, (5.00 g, 5.65 mmol), and 9,9-bis(4-aminophenyl)fluorene (1.97 g, 5.65 mmol) in DMSO (120 mL). Data for polymer **I**: IR (neat): ν (cm^−1^) 3060, 1616, 1596, 1546, 1495, 1448, 1395, 1359, 1214, 1181, 1119, 1032, 1011, 842, 816, 766, 738, 700, 678, 561. δ_H_ (*d*_6_-DMSO, 400 MHz, ppm): 8.81 (4H, s, aromatic meta to N^+^), 7.85–7.87 (2H, d, *J* = 7.6 Hz, aromatic), 8.62 (4H, s, 1,4-phenylene ring between pyridinium rings), 7.31–7.44 (32H, m, aromatic), 7.04–7.05 (4H, d, *J* = 7.6 Hz, tosylate), 6.95–6.97 (2H, d, *J* = 6.4 Hz, aromatic), 6.45–6.47 (4H, d, *J* = 7.2 Hz, tosylate), 2.23 (6H, s, CH_3_). δ_C_ (*d*_6_-DMSO, 100 MHz, ppm): 156.87, 156.83, 154.44, 146.24, 137.93, 133.32, 130.37, 129.22, 128.43, 127.83, 127.83, 127.81, 125.91, 21.22. Anal. calcd. for C_79_H_58_N_2_O_6_S_2_ (1195.45): C, 79.37; H, 4.89; N, 2.34; S, 5.36. Found: C, 77.76; H, 4.94; N, 2.37; S, 5.17.

### 2.5. Synthesis of Polymers ***II***–***V***

Polymers **II**–**V** were prepared by the metathesis reaction of polymer **I** with the respective excess of salts of appropriate counterions in a common organic solvent such as acetonitrile [2]. The procedure that was employed is described as follows. Two grams (1.67 mmol) of polymer **I** were dissolved in 50 mL of acetonitrile on gentle warming. To this polymer solution, 2.4 g (8.4 mmol) of lithium triflimide in 50 mL of acetonitrile were added dropwise on stirring. The resulting solution was kept at 50 °C overnight with stirring. After removing acetonitrile completely by a rotary evaporator, distilled water was added to the solid products to dissolve both lithium tosylate and excess lithium triflimide, affording the desired polymer **II**. It was collected by filtration, washed several times with a large quantity of distilled water, dried in vacuum at 50 °C for 48 h, and weighed to give 2.1 g (1.49 mmol) of polymer **II** (yield 89%).

Data for polymer **II**: IR (neat): ν (cm^−1^) 3064, 1598, 1577, 1545, 1496, 1449, 1347, 1228, 1178, 1131, 1055, 1001, 918, 842, 821, 779, 763, 738, 699, 674, 650, 598, 570, 508. δ_H_ (*d*_6_-DMSO, 400 MHz, ppm): 8.85 (4H, s, aromatic meta to N^+^), 8.64 (4H, s, 1,4-phenylene ring between pyridinium rings), 7.86–7.88 (2H, d, *J* = 8.0 Hz, aromatic), 7.32–7.41 (30H, m, aromatic), 6.95–6.96 (2H, d, *J* = 4.8 Hz, aromatic), 6.49–6.51 (4H, d, *J* = 7.2 Hz, aromatic). δ_C_ (*d*_6_-DMSO, 100 MHz, ppm): 156.89, 133.25, 130.37, 130.31, 130.24, 130.18, 130.15, 129.15, 128.48, 128.43, 128.38, 127.85, 125.98, 125.95, 121.52, 118.32. δ_F_ (*d*_6_-DMSO, 376 MHz, ppm): −78.75. Anal. calcd. for C_69_H_44_N_4_O_8_F_12_S_4_ (1413.35): C, 58.64; H, 3.14; N, 3.96; S, 9.07. Found: C, 58.69; H, 3.14; N, 3.98; S, 8.86.

Data for polymer **III**: IR (neat): ν (cm^−1^) 3058, 2957, 2928, 2858, 1728, 1618, 1598, 1577, 1497, 1448, 1391, 1314, 1222, 1156, 1086, 1035, 1031, 918, 844, 823, 766, 738, 729, 701, 628, 608, 564, 519. δ_H_ (CD_3_OD, 400 MHz, ppm): 8.60 (4H, s, aromatic meta to N^+^), 8.44 (4H, s, 1,4-phenylene ring between pyridinium rings), 7.78–7.80 (2H, d, *J* = 7.6 Hz, aromatic), 7.32–7.43 (26H, m, aromatic), 7.16–7.18 (4H, d, *J* = 7.6 Hz, aromatic), 6.92–6.94 (2H, d, *J* = 6.8 Hz, aromatic), 6.68–6.70 (4H, d, *J* = 7.2 Hz, aromatic), 3.95–3.99 (11H, m, AOT), 3.07–3.15 (2H, m, AOT), 2.94–2.95 (2H, m, AOT), 1.52–1.55 (5H, m, AOT), 1.27 (37H, m, AOT), 0.84–0.88 (26H, m, AOT). δ_C_ (CD_3_OD, 100 MHz, ppm): 171.14, 168.21, 157.13, 137.43, 132.86, 130.13, 129.68, 128.60, 128.13, 127.82, 125.94, 67.28, 67.22, 66.23, 66.69, 61.89, 38.73, 38.70, 38.67, 38.62, 33.52, 30.06, 29.96, 29.93, 28.65, 28.62, 23.40, 23.37, 23.28, 23.25, 22.60, 13.05, 13.01, 9.96. Anal. calcd. for C_105_H_118_N_2_O_14_S_2_ (1696.20): C, 74.35; H, 7.01; N, 1.65; S, 3.78. Found: C, 73.71; H, 7.13; N, 1.78; S, 3.62.

Data for polymer **IV**: IR (neat): ν (cm^−1^) 3062, 1600, 1517, 1497, 1447, 1363, 1197, 1142, 1113, 1035, 1029, 1007, 944, 842, 822, 767, 740, 729, 692, 640, 619, 571. δ_H_ (*d*_6_-DMSO, 400 MHz, ppm): 8.80 (4H, s, aromatic meta to N^+^), 8.60 (4H, s, 1,4-phenylene ring between pyridinium rings), 7.75–7.87 (16H, m, aromatic), 7.67–7.68 (28H, m, aromatic), 7.33 (2H, s, aromatic), 6.78–6.98 (4H, m, aromatic), 6.45–6.47 (3H, m, aromatic), 3.02 (12H, m, CH_3_ MO). δ_C_ (*d*_6_-DMSO, 100 MHz, ppm): 156.83, 152.97, 152.53, 149.55, 143.00, 139.92, 138.12, 137.14, 133.32, 130.31, 130.20, 129.20, 128.47, 128.35, 127.85, 126.95, 125.94, 125.22, 121.60, 111.98. Anal. calcd. for C_79_H_58_N_8_O_6_S_2_ (1461.75): C, 76.42; H, 4.96; N, 7.67; S, 4.39. Found: C, 74.47; H, 5.25; N, 7.25; S, 4.07.

Data for polymer **V**: IR (neat): ν (cm^−1^), 3060, 2973, 2924, 2872, 1619, 1598, 1577, 1499, 1454, 1345, 1332, 1196, 1056, 1009, 828, 766, 738, 729, 701, 655, 628. δ_H_ (CD_3_OD, 400 MHz, ppm): 8.57 (4H, s, aromatic meta to N^+^), 8.42 (4H, s, 1,4-phenylene ring between pyridinium rings), 7.80–7.90 (2H, m, aromatic), 7.67–7.78 (4H, m, aromatic), 7.30–7.41 (27H, m, aromatic), 7.16–7.18 (4H, d, *J* = 8.0 Hz, aromatic), 6.92–6.94 (2H, d, *J* = 7.2 Hz, aromatic), 6.68–6.70 (3H, m, AOT), 0.72–1.56 (55H, m, AOT). δ_C_ (CD_3_OD, 100 MHz, ppm): 157.13, 132.84, 130.12, 129.65, 128.59, 128.11, 127.82, 127.81, 125.89, 125.23, 125.31, 125.26, 78.04, 28.42, 28.37, 28.13, 28.10, 28.06. Anal. calcd. for C_101_H_102_N_2_O_6_S_2_ (1504.30): C, 80.66; H, 6.84; N, 1.86; S, 4.26. Found: C, 79.36; H, 6.70; N, 1.99; S, 4.05.

### 2.6. X-Ray Scattering Experiments

SAXS experiments were conducted using the CREDO facility of the Hungarian Research Centre for Natural Sciences [28,29]. Cu Kα X-rays were produced by a GeniX3D Cu ULD integrated beam delivery system (Xenocs SA, Sassenage, France) with the X-ray wavelength 0.154 nm. The scattered radiation was detected using a Pilatus-300k CMOS hybrid pixel position sensitive detector (Dectris Ltd., Baden, Switzerland) placed 536 mm downstream from the sample. The accessible *q*-range was 0.2 to 5.3 nm^−1^ calibrated by SBA15mesoporous silica and silver behenate.

The samples were put into borosilicate glass capillaries of approx. 2 mm outer diameter and 0.01 mm wall thickness and kept at room temperature. Exposures of each sample were repeated in 5 min units with frequent re-measuring of background signals and calibration samples until the desired signal-to-noise ratio was obtained. Each scattering pattern was corrected for sample self-absorption, instrumental background, and detector flatness using the standard procedure implemented in the data collection program. The scattering patterns were statistically filtered to remove artefacts from external radiation. The scattering intensity $I\left(q\right)$ was transformed to absolute units (differential scattering cross section) by measuring a glassy carbon sample of known absolute scattering intensity under the same conditions as our samples.

Initial data interpretation was made using a simple scaling argument. In this consideration, $I\left(q\right)$ follows a power law $I\left(q\right)\propto q^{-\alpha }$ where the exponent α=4 refers to 3-dimensional particles with smooth surface (Porod’s law) and α=1 refers to rod-like particles.

When the sample was interpreted as a “dense network” of rod-like particles, the data were fitted to the equations:

(1) $$I\left(q\right)\propto \frac{1}{{\left(1+\xi_{s}^{2}q^{2}\right)}^{2}},\;\mathrm{and}$$

(2) $$I\left(q\right)\propto \frac{1}{1+q\xi_{d}\exp\left(\frac{q^{2}R^{2}}{4}\right)}$$

where the first equation represents Debye–Bueche equation and the second an represents Ornstein–Zernike-type equation. Here, $\xi_{s}$ represents a typical distance within the two-phase network, $\xi_{d}$ represents a mesh size in the network, and *R* represents the radius of a rod-like particle, ultimately a rod-like polymer. This model and its applicability to LC π-conjugated polymers was discussed for details [30,31,32,33].

When the sample was interpreted as a “gel” with a clear Guinier plateau, the data were fitted to the equations:

(3) $$I\left(q\right)\propto \exp\left(-\frac{q^{2}R_{g}^{2}}{3}\right),\;\mathrm{and}$$

(4) $$I\left(q\right)\propto \frac{1}{{\left(1+[\frac{\left(D+1\right)}{3}]\xi^{2}q^{2}\right)}^{\frac{D}{2}}}$$

where the first equation represents Guinier equation. The second equation is a modified Ornstein–Zernike-type equation that reduces to the Ornstein–Zernike equation aka Lorentz equation for D=2. Here, $R_{g}$ is a longer distance correlation length and ξ is a shorter distance correlation length, whereas D represents the fractal dimension deduced from the data decay. This model and its applicability to gel-like polymer systems was discussed for details [34,35].

When the data showed a −4 slope with an emerging plateau at lower scattering angles, the data were fitted to the generalized Guinier law:

(5) $$I\left(q\right)\propto \exp\left[-\frac{q^{2}R_{q}^{2}}{3-s}\right]$$

where $R_{g}$ represents a radius of gyration or simply some longer distance correlation length and where 3−s is a dimensionality parameter with s=2 for rod-like particles for details [36].

When the data showed a −4 slope with an emerging maximum at lower scattering angles, the data were fitted to the Teubner–Strey equation:

(6) $$I\left(q\right)\propto \frac{1}{a_{2}+c_{1}q^{2}+c_{2}q^{4}}$$

where the parameters $a_{2}$, $c_{1}$, and $c_{2}$ are defined in terms of periodicity and correlation length for details [37].

## 3. Results and Discussion

### 3.1. Chemical Structures of Polymers ***I***–***V***

Polymer **I** was prepared by the ring-transmutation polymerization reaction of bispyrylium salt, **M**, and 9,9-bis(4-aminophenyl) fluorene that was performed on heating in dimethyl sulfoxide at 140–150 °C for 24 h under a blanket of nitrogen [1]. To increase the molecular weight of the polymer, a few mL of toluene were added to the reaction flask to remove the generated water during the polymerization reaction by a Dean–Stark trap, while the polymers **II**–**V** were made by a metathesis reaction of polymer **I** with the corresponding sodium or lithium organic salts in a common organic solvent such as acetonitrile, as outlined in Scheme 1. The chemical structures of polymers **I**–**V** were confirmed by FTIR, ^1^H, ^13^C, and ^19^F (when applicable) NMR spectra and elemental analyses. The FTIR spectrum (Figure S1) showed the representative characteristic peaks for polymer **I**: 1620–1448 (C=C and C=N aromatic ring stretching), 1196 (C–N^+^), 1119 (S=O asymmetric stretching), and 1033–1010 (S=O symmetric stretching). After an exchange of tosylate to triflimide, an additional C–F stretching vibration at ν = 1350 cm^−1^ in polymer **II** (Figure S2) was observed, indicating the presence of a triflimide counterion. Moreover, polymers **III** and **IV** (Figures S3 and S4) displayed unique additional peaks at ν = 1736 cm^−1^ (C=O stretching) from a dioctyl sulfosuccinate counterion and 1458 and 1365 cm^−1^ (N=N and C–N stretching) from methyl orange, respectively. Polymer **V** showed the expected absorptions peaks (Figure S5). The ^1^H NMR spectrum of polymer **I** (Figure S6) showed characteristic broad peaks at δ = 8.82 and 8.62 ppm for the protons of the aromatic moieties of poly(pyridinium salt) and a set of resonances at δ = 7.45, 7.08 and 2.27 ppm for the protons of the aromatic moiety and methyl group in the tosylate counterion. The exchange of a counterion from tosylate to triflimide was confirmed by the disappearance of tosylate resonances and the appearance of a new signal at δ = −78.75 ppm in the ^19^F NMR spectrum (Figure S7), resulting in the completion of the reaction. For polymer **III** (Figure S8), new additional peaks from the dioctyl sulfosuccinate counterion appeared in the range of the aliphatic region and their ^1^H integral ratios were in good agreement with one another. After exchanging the counterion from tosylate to methyl orange, a new set of signals appeared at δ = 7.79, 7.70, 6.80 (aromatic protons), and 3.06 (–N(CH_3_)_2_) in the ^1^H NMR spectrum of **IV** (Figure S9). The polymer **V**, which had dodecylbenzene sulfonate as the counterion, showed the expected proton signals in the ^1^H NMR spectrum (Figure S10). All these results indicated the metathesis reaction went smoothly to completion without causing any side reactions. Their ^13^C NMR spectra also showed the expected carbon signals (Figures S6–S10). All FTIR, ^1^H, ^13^C, and ^19^F (when applicable) spectra of polymers **I**–**V** are provided in the Supplementary Materials (Figures S1–S10).

### 3.2. Polyelectrolyte Behavior of Polymers ***I*** and ***II***

**Figure 1:**
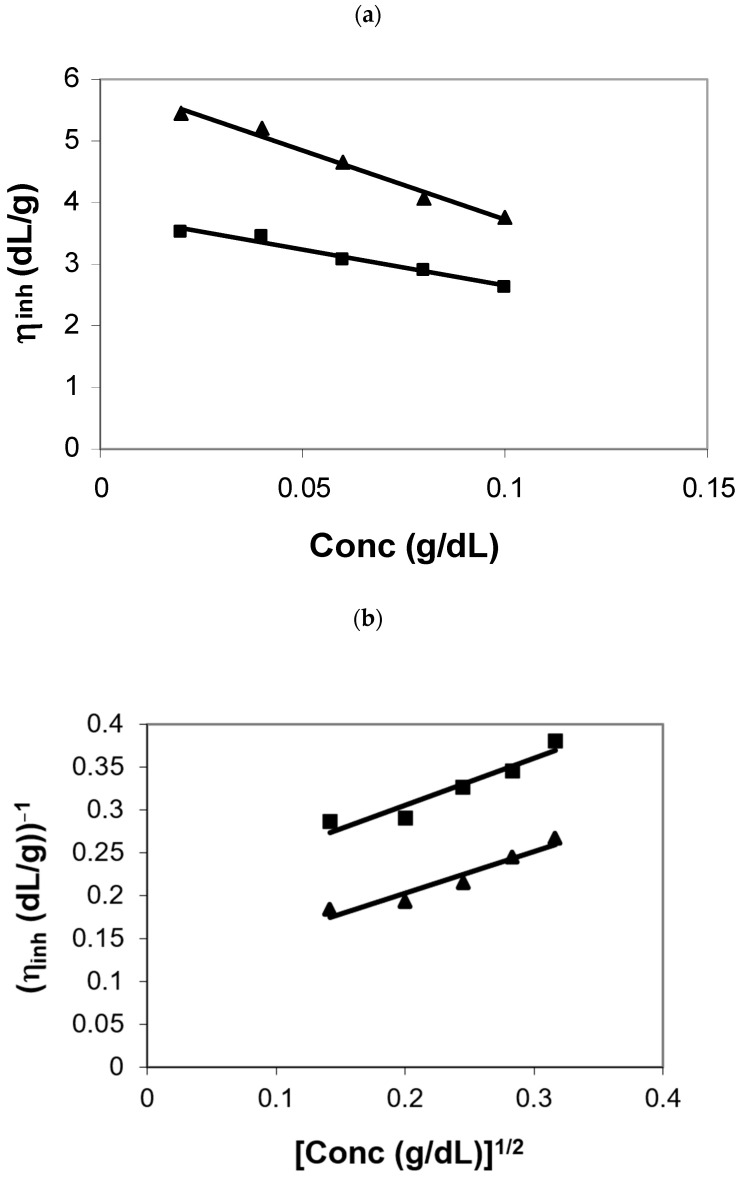
(**a**) Polyelectrolyte behavior of polymers **I** (▲) and **II** (■) in DMSO at 35 °C; (**b**) Fuoss plots of polymers **I** (▲) and **II** (■).

Polymers **I**–**V** exhibited a polyelectrolyte behavior in DMSO, since they contained the ionic groups of 4,4′-(1,4-phenylene) bis (2,6-diphenylpyridinium) ions along the backbones of the polymer chains. This behavior is the signature of ionic polymers in either organic solvents or water depending on their chemical structures. For example, polymers **I** and **II** showed a polyelectrolyte behavior in DMSO for a concentration range of 0.02–0.10 dL/g; that is, the inherent viscosity (IV) values of polymer solutions increased with the decrease in polymer concentrations (Figure 1a) [38,39,40,41]. As expected, they obeyed the empirical Fuoss equation [38], which is usually applied to random coiled polyelectrolytes. The Fuoss equation is written as follows:

(7) $$\eta_{inh}=\frac{A}{1+{BC}^{0.5}}\Rightarrow \;\;\;\;\;\frac{1}{\eta_{inh}}=\frac{1}{A}+\frac{B}{A}C^{0.5}$$

where η_inh_ and *C* are usual notations while *A* and *B* are constants.

Despite the presence of an sp^3^-C linkage in the fluorene moiety along the backbone of these polymers, their intrinsic viscosity values obtained from the intercepts of Fuoss plots (Figure 1b) were 9.57 and 5.12 dL/g in DMSO at 35 °C, respectively, indicative of their relatively high molecular weights. Furthermore, their finger-nail creasable film, forming from several common organic solvents that included methanol, acetonitrile, and DMSO, was also indicative of their high molecular weights, which enabled us to eliminate the effect of molecular weights on the lyotropic and photophysical properties of these polymers (vide *infra*).

### 3.3. Molecular Weight of Polymer ***I*** by Gel Permeation Chromatography (GPC)

Measurements of molecular weights of ionic polymers by GPC are indeed challenging tasks, since the ionic groups cause aggregation and ionic interactions between the column packaging materials of the instrument. These ionic interactions are suppressed by the addition of 0.01 M LiBr in the chosen solvent. The molecular weight of polymer **I** was estimated in terms of dynamic light scattering (DLS) using Malvern Zetasizer Nano. Several samples with their concentrations ranging from 0.25 to 5 mg/mL dissolved in DSMO were measured at room temperature with and without shape correction using the Rayleigh Equation and Debye plots. By using this instrument, the weight-average molecular weight (M_w_) of polymer **I** was found to be 176 kg/mol, which indicated this polymer had a high molecular weight. It was expected other polymers **II**–**V** had similar ranges of molecular weights, since they were synthesized by the metathesis reactions of polymer **I** with the corresponding salts in acetonitrile. It is reasonable to assume that their solution properties including lyotropic and optical properties can be further studied without any concerns for the secondary effects of molecular weights on these polymers.

### 3.4. Lyotropic Properties of Polymers ***I***–***V*** by Polarized Optical Microscopy (POM)

**Figure 2:**
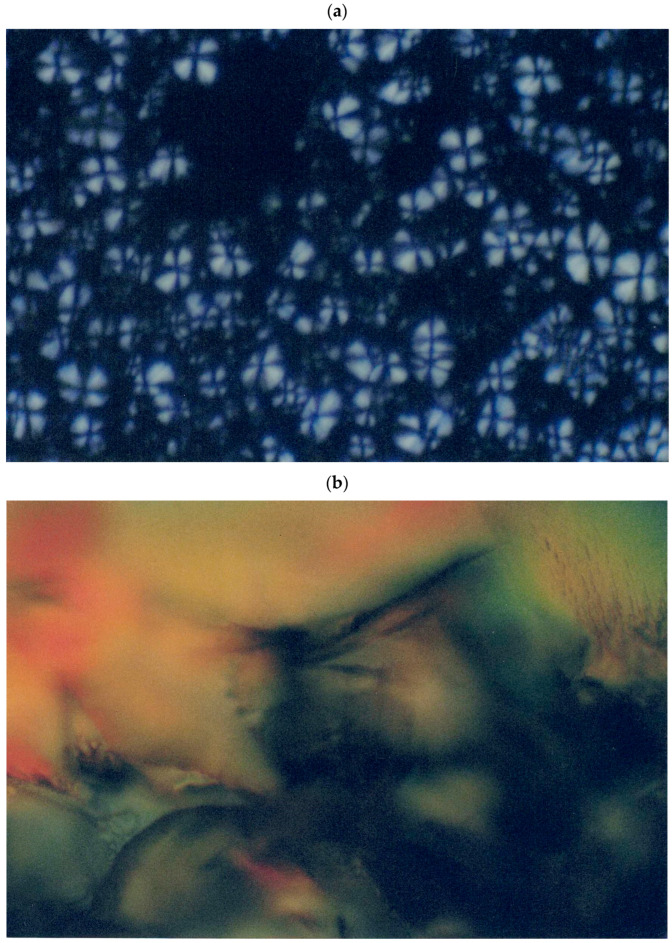
(**a**) Photomicrograph of (**a**) polymer **I** at 20 wt% in methanol and (**b**) polymer **II** at 61 wt% in acetonitrile under crossed polarizers taken at room temperature exhibiting biphasic and lyotropic liquid-crystalline (LC), respectively (magnification 400×).

The solution properties including the lyotropic properties of **I–V** are collected in Table 1. Figure 2 shows the biphasic solution of polymer **I** at 20 wt% in the methanol and lyotropic phase of polymer **II** at 61% in acetonitrile. Polymer **I** did not form a lyotropic phase in acetonitrile because it did not exhibit enough solubility in this solvent. Similarly, polymer **II** did not form a lyotropic phase in methanol because of insufficient solubility. Note here that polymer **II** had enough solubility in DMSO to form a lyotropic phase, but its lyotropic phase could not be identified by the POM studied, since it formed very viscous solutions that precluded the identification of a lyotropic phase. Polymer **III** formed a lyotropic phase at 72 wt% in DMSO and at 67 wt% acetonitrile because of strong interactions of this polymer with these solvents. Polymer **IV** exhibited a lyotropic phase at 55 wt% in DMSO only, since it had suitable and sufficient interactions with this solvent. However, it had poor interactions with acetonitrile and methanol to preclude the formation of a liquid-crystalline phase. On the other hand, polymer **V** formed a lyotropic phase at 50 wt% in DMSO, at 67 wt% in acetonitrile, and at 63 wt% in methanol (Table 1). Therefore, it was found that the relatively high concentrations of polymer solutions for the formation of lyotropic phases were related to the nonlinear tetrahedral carbon center fluorene moieties that increased the solubility of these polymers considerably. Generally, several key factors are responsible for the formation of the lyotropic phases in a polymer including rod-like structures with an extended chain structure to facilitate the alignment of the polymer chain along a particular direction. Another factor is the sufficient solubility to exceed the critical concentration of a polymer. The solubility and chain stiffness of a polymer are dependent on the microstructure, molecular weight, polymer–polymer and polymer–solvent interactions, and temperature [1,5,42]. Note here that at a low concentration, a polymer forms an isotropic solution in each solvent. At an intermediate concentration, a polymer forms a biphasic solution in which there exists a liquid-crystalline phase and an isotropic phase. At a relatively high concentration, a polymer forms a fully grown lyotropic phase wherein this phase occurs with the further addition of a polymer at the expense of an isotropic phase of a biphasic solution.

### 3.5. Lyotropic Properties of Polymers ***I*** and ***II*** by Small Angle X-Ray Scattering (SAXS)

**Figure 3:**
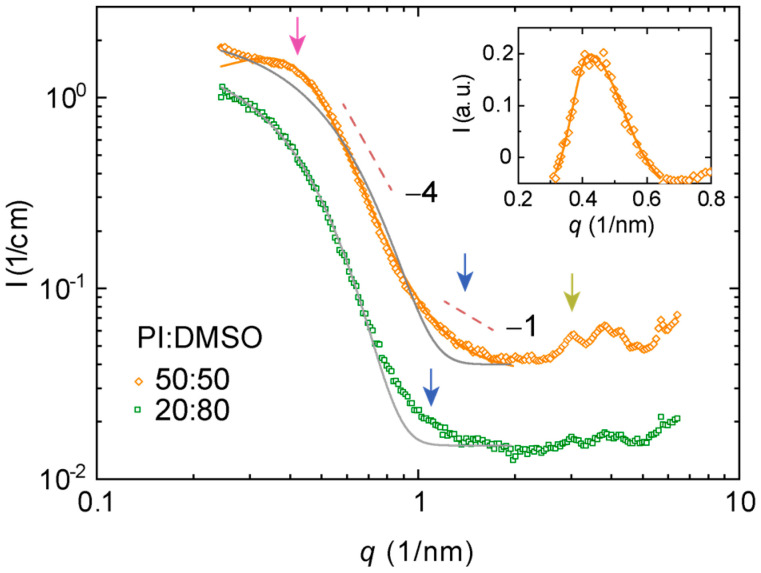
SAXS patterns of polymer **I** in DMSO. The employed mixing ratios of polymer:solvent were 50:50 (orange diamonds) and 20:50 (green squares). Orange solid line is a fit to Teubner–Strey model. Dashed terracotta lines illustrate −4 and −1 decays for comparison. Gray solid lines show generalized Guinier–Porod functions for comparison. Arrows mark a characteristic maximum at *q* ≈ 0.42 1/nm (magenta), minus one decay (blue), and Bragg reflections (dark yellow). Inset shows the residual from the maximum when the Guinier–Porod function was deducted as a phenomenological background.

**Figure 4:**
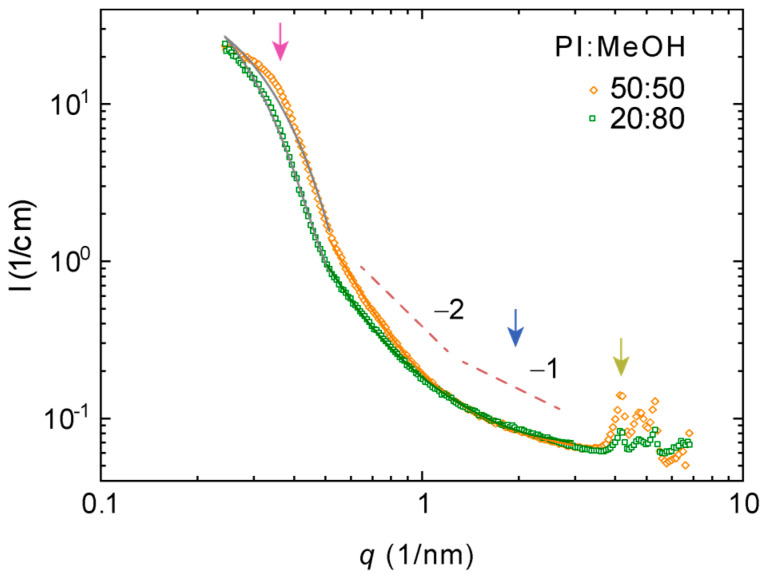
SAXS patterns of polymer **I** in methanol. The employed mixing ratios of polymer:solvent were 50:50 (orange diamonds) and 20:50 (green squares). Solid orange and green lines are the fits to the combined Debye–Buche/Ornstein–Zernike model. Dashed terracotta lines illustrate −2 and −1 decays for comparison. Gray solid lines show Guinier functions for comparison. Arrows mark a characteristic maximum (magenta), minus one decay (blue), and Bragg reflections (dark yellow).

Figure 3 and Figure 4 show characteristic SAXS patterns of polymer **I** in DMSO and methanol. Parameters estimated from the fits to these data are compiled in Table 2. All the materials studied appear as dense fluids. Both samples are interpreted as lyotropic based on polarized microscopy. The data deviated from the generic Guinier–Porod model describing elongated scatterers in matrix. Both systems showed a broad interference maximum at a lower *q*-range for high polymer concentrations (marked by magenta arrows). This points to a larger length-scale order, which is typical for dense polyelectrolytes and lyotropic LCs. This might be understood in terms of a two-phase material with two length scales—a correlation length and a domain size fitted to the Teubner–Strey model (Figure 3). Characteristic for all data is −1 decay within some *q*-range, which we attributed to the locally isolated rigid polymers or polymer segments typical for conjugated polymer backbones in dissolutions (see blue arrows). This means that the sample may be interpreted as a “dense network” of rod-like particles and fitted to the combinations of Debye–Bueche and Ornstein–Zernike-type equations (Figure 4). This is another two-phase model discussed in detail elsewhere for LC polyfluorenes [30,31]. At the same time, all datasets showed a set of Bragg reflections (marked by dark yellow arrows) pointing to emerging crystal growth or a polymer fraction that was not properly mixed. We tentatively found a peak sequence corresponding to the cubic order akin to our previous results with lyotropic poly(pyridinium salts) [5] or thermotropic poly(2,5-pyridinium methane sulfonates) [6] or lamellae order observed in other thermotropic poly(2,5-pyridinium sulfonates) [43] but the data quality did not allow proper crystallographic analysis.

**Figure 5:**
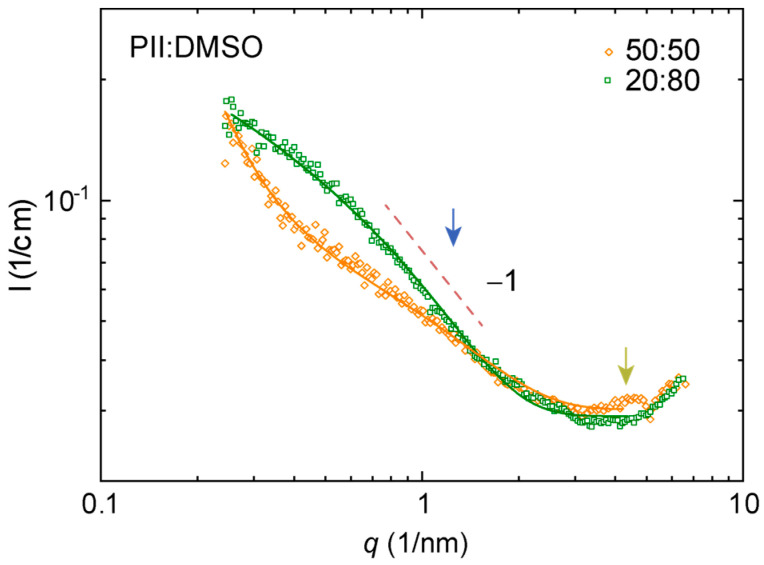
SAXS patterns of polymer **II** in DMSO. The employed mixing ratios of polymer:solvent were 50:50 (orange diamonds) and 20:50 (green squares). Solid orange and green lines are the fits to the combined Debye–Buche/Ornstein–Zernike model. Dashed terracotta line illustrates −1 decay for comparison. Arrows mark a characteristic minus one decay (blue) and emerging Bragg reflections (dark yellow).

Figure 5 shows characteristic SAXS patterns of polymer **II** in DMSO. It was not clear whether this sample was lyotropic. This dataset had certain similarities with that of polymer **I** but not as distinctive. Again, we observed a −1 decay attributed to the locally isolated polymer segments (marked by a blue arrow). Similarly, it was possible to fit a Debye–Bueche/Ornstein–Zernike model where the denser case showed an upturn as expected for the model in the first place even though the intensity was lower in the middle *q*. Faint Bragg reflections, akin to Figure 3 and Figure 4, were also visible for a higher polymer concentration (marked by a dark yellow arrow).

**Figure 6:**
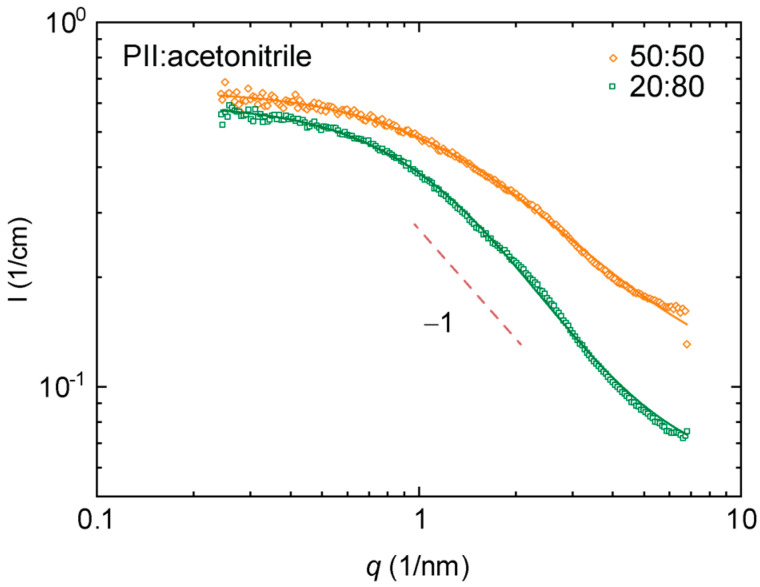
SAXS patterns of polymer **II** in acetonitrile. The employed mixing ratios of polymer:solvent were 50:50 (orange diamonds) and 20:50 (green squares). Solid orange and green lines are the fits to the gel model.

Figure 6 shows characteristic SAXS patterns of polymer **II** in acetonitrile. The parameters estimated from the fits to the data are listed in Table 2. This sample was deemed as lyotropic but we noted that the optical appearance of polymer **II** in acetonitrile differed from polymer **I** in DMSO and methanol (see Figure 1 and Figure 2). The scattering curves deviated systematically from those shown in Figure 3, Figure 4 and Figure 5 and from those shown in Reference [5]. These data are reminiscent of the SAXS data of poly(2,5-pyridinediyl) complexed with camphor sulphonic acid mixed in formic acid [43], but these were observed for lower concentrations and interpreted as Debye chains. We observed that the data followed the model proposed for polymer distribution in a gel with two characteristic length scales.

### 3.6. Optical Properties of Polymers ***I***–***V*** by UV-Vis and Fluorescence Spectrometers

The optical properties of the poly(pyridinium salt)-fluorenes **I**–**V** containing different counterions were studied in methanol at 1.0 × 10^−5^ M concentration (except for polymers **II** and **IV**), and their molar absorptivities were also calculated and are compiled in Table 3. While fully π-conjugated polyelectrolytes possess the unique capacity to display optical properties, they are typically not strong due to aggregation-induced quenching effects in solution. The outstanding solubility properties of these polymers enabled the study of their photoluminescent properties in organic solvents. Polymer **II** was not soluble in methanol up to 1.0 × 10^−4^ M and, unfortunately, polymer **IV** containing the methyl orange counterion did not possess sufficient solubility in this solvent for its optical properties to be studied. The analogous polymers that incorporated the 9,9′-dioctylfluorene moieties in the polymer backbones demonstrated solubilities in solvents such as chloroform and tetrahydrofuran, which indicated that a combination of an organic counterion and n-octyl chain substituents dramatically increased the solubility of the polymer [5].

**Figure 7:**
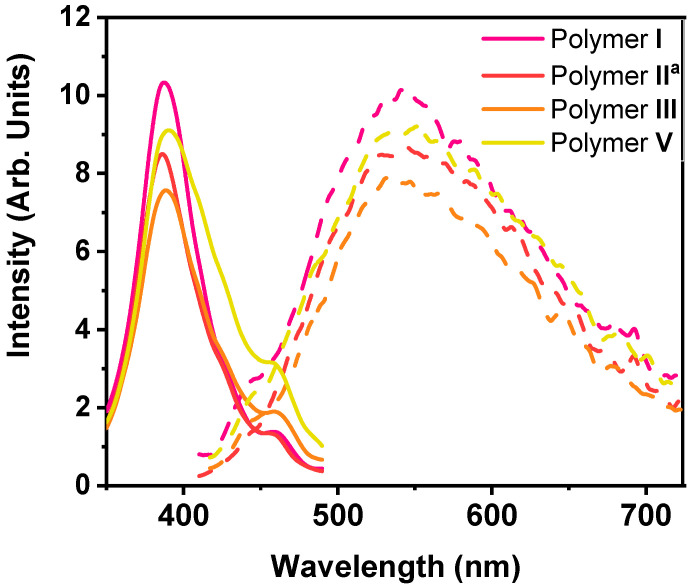
Emission spectra (right, dotted lines) and excitation spectra (left, solid lines) of polymers **I**, **II^a^**, **III**, and **V** in methanol (^a^ measured in acetonitrile).

For the polymers investigated, the change in counterion structure did not affect the optical properties, as suggested for the data in Table 3. The UV-Vis spectra (Figures S11–S14) for all polymers had maximal absorption at around λ_abs_ = 340 nm. Their relatively high molar absorptivity values were in great agreement with previously reported poly(pyridinium salt)s that ranged from 57,000–66,000 M^−1^ cm^−1^ [5,44]. Compared to the previously studied poly(pyridinium salt)-fluorenes containing the flexible 9,9′-dioctylfluorene moiety, they had a slight hypsochromic shift [5]. In contrast, their emission values ranged from λ_em_ = 534–541 nm (Figure 7 and Figures S15–S18), which was bathochromically shifted in comparison to the previous polymers but in the range for other poly(pyridinium salt)s in the literature [5,44]. Additionally, their emitted values ranged from 565–599 nm in DMSO. The absolute quantum yield (AQY), Φ_F_, values for the polymers in solution were found to be low, in the range of 0.022–0.027, which were still much higher than the relative quantum yield (0.0088) of conjugated poly(pyridinium salt) based on the benzidine moiety measured in N,N-dimethylformamide [44,45]. These polymers had good light-emitting properties and emitted greenish-yellow lights in methanol or acetonitrile. In the solid state, AQY values were in the identical range, of Φ_F_ = 0.020–0.034, to that of solutions AQYs. Polymer **II** also exhibited negligible fluorescence in the solid state, suggesting that the bistriflimide anion plays a role in quenching the fluorescence of the polymer in the solution and solid state.

### 3.7. Emission Properties of Polymers ***I***, ***II***, ***III***, and ***V*** in Organic Solvents–Water Mixture

**Figure 8:**
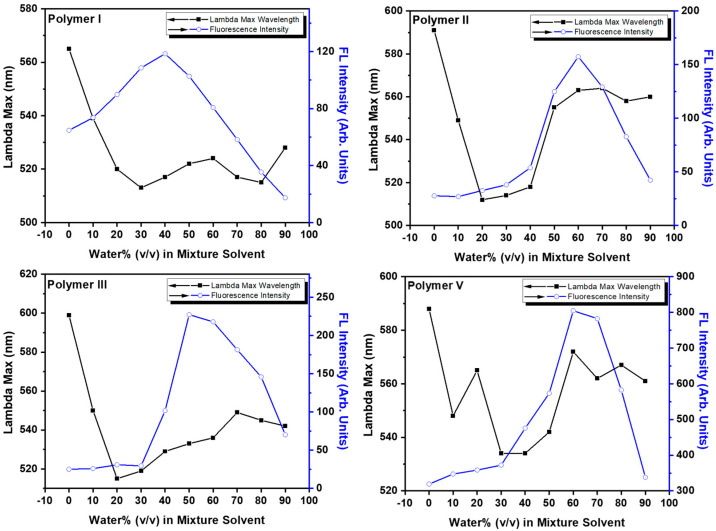
Fluorescence intensity and emission peak of polymers **I**, **II**, **III**, and **V** as a function of water content in DMSO (1 μM repeating units, λ_ex_ at 390 nm).

Water-induced aggregation effects, which impacted the fluorescence of the polymers, were studied in various organic solvent–water mixtures to understand behavior in aqueous media. DMSO/H_2_O (*v*/*v*, 0–90%) provided the best results for augmenting fluorescence (Figure 8) because the viscous nature of DMSO encourages the inhibition of nonradiative decay in the excited state of a polymer [5,46]. For polymers **II**, **III**, and **V**, the emission intensity peaked at around 60% (*v*/*v*) and for polymer **I,** it peaked at 40%. All polymers studied exhibited a steady increase in fluorescence intensity from around 0% to 40–60% (*v*/*v*) followed by a steady decrease beyond the peak intensity (60 to 90% (*v*/*v*)). It did not follow the complex fluorescence intensity observed in the poly(pyridinium salt)-fluorenes containing the flexible 9,9′-dioctylfluorene moiety, where relatively erratic changes in intensity were observed with each addition of poor solvent (water) [5]. Polymer precipitation was not observed for any solutions, even at 90% (*v*/*v*). All these polymers displayed a dramatic hypsochromic shift from 0 to 20% (*v*/*v*), except polymer **V,** which was less shifted and even increased in λ_em_ wavelength at 20% (*v*/*v*). Polymers **I** and **III** increased in λ_em_ wavelength beyond 20% (*v*/*v*), a small amount from around 520 to 535 nm compared to polymers **II** and **V,** which had a considerably larger bathochromic shift from around 520 to 560 nm.

Additional organic solvent–water systems were also studied for these polymers. Figures S19, S21 and S23 show the spectra of polymers **I**, **III**, and **V** in CH_3_OH/H_2_O (*v*/*v*, 0–90%), respectively. Polymer **I** did not appear to possess aggregation-induced emission effects from water addition as there was a steady decrease in fluorescence intensity with the added volume of water; however, there was a dramatic bathochromic shift in λ_em_ starting at 70% (*v*/*v*) from about 460 to 560 nm. Polymers **III** and **V** increased in fluorescence intensity starting at 50% (*v*/*v*), with similar behavior in the DMSO system. Bathochromic shifts were also observed for them, where polymer **III** started λ_em_ shifting at 70% (*v*/*v*) and polymer **V** started λ_em_ shifting at 50% (*v*/*v*), but interestingly they started exhibiting hypsochromic shifts starting at 90% (*v*/*v*) and 60% (*v*/*v*), respectively. The CH_3_CN/H_2_O (*v*/*v*, 0–90%) system was also studied for polymers **II**, **III**, and **V,** as shown in Figures S20, S22 and S24, respectively. Polymer **I** was not soluble enough in acetonitrile to perform this study. Polymer **II** sharply increased in fluorescence intensity at 70% (*v*/*v*) and then decreased at 90% (*v*/*v*). A bathochromic shift to 590 nm was also observed starting at 60% (*v*/*v*) followed by a decrease to 580 nm at 90% (*v*/*v*). Polymers **III** and **V** steadily decreased in fluorescence intensity from around 0% to 70% (*v*/*v*) but started increasing from 70% to 90% (*v*/*v*); for polymer **III**, it was a considerably higher increase in fluorescence intensity. They also followed the same trend in bathochromically shifting starting at 60% (*v*/*v*) and then decreasing at 90%. Emission spectra of all polymers with varying amounts of water are included in the Supplementary Materials (Figures S25–S34).

## 4. Conclusions

Five poly(pyridinium salt)-fluorene polymers containing 9,9-bis(4-aminophenyl)fluorene moieties in the backbone of the polymer chains with various organic counterions were prepared by using ring-transmutation and metathesis reactions. Their chemical structures were characterized by FTIR, ^1^H, ^13^C, and ^19^F NMR (whenever applicable) spectroscopic techniques, and elemental analysis. They showed polyelectrolyte behavior in DMSO because of the positive charges along the backbone of the polymer chains. Their lyotropic liquid-crystalline phases in organic solvents (DMSO, CH_3_CN, or MeOH) were examined by POM and SAXS studies. Polymer **I** exhibited lyotropic liquid-crystalline phases in DMSO and CH_3_OH; polymer **II** and polymer **III** exhibited in DMSO and CH_3_CN; polymer **IV** exhibited in DMSO only; and polymer **V** exhibited in all three polar organic solvents. Some of them had good light-emitting properties and emitted green lights in methanol or acetonitrile. In the solid state, some AQY values (Φ_F_) were in the identical range of 0.020–0.034 to that of solutions AQYs. Additionally, they formed aggregated structures in DMSO, CH_3_CN, and CH_3_OH with the addition of water (*v*/*v*, 0–90%) regardless of the chemical structures of the counterions, and the emission peaks of these aggregated structures were blue shifted.

## Figures and Tables

**Table 1:** Solution properties of poly(pyridinium salt)s−fluorene, **I**–**V**, in different organic solvents.

	Polymer				
Solvent	**I**	**II**	**III**	**IV**	**V**
DMSO	1–10 wt% I ^a^	-	1–54 wt% I	1–54 wt% I	1–54 wt% I
	21 wt% B ^a^	^b^	62 wt% B	62 wt% B	62 wt% B
	30 wt% L ^a^	-	72 wt% L	72 wt% L	72 wt% L
CH_3_CN	-	-	1–40 wt% I	-	40 wt% I
	-	50 wt% L	67 wt% L	-	54 wt% B
	-	-	-	-	67 wt% L
CH_3_OH	10 wt% B	-	-	-	49 wt% I
	30 wt% L	-	-	-	63 wt% L

^a^ I = isotropic; B = biphasic (anisotropic + isotropic); and L = lyotropic (birefringence). ^b^ It is too difficult to detect I, B, and lyotropic phases by POM.

**Table 2:** Structural parameters estimated from the selected fits to the SAXS patterns (n/a = not applicable).

	PI/MeOH		PII/DMSO		PII/Acetonitrile	
	20:80	50:50	20:80	50:50	20:80	50:50
$\xi_{s}\left(\mathrm{nm}\right)$	21.4 ± 0.1	18.4 ± 0.1	n/a	8.9 ± 0.1	n/a	n/a
$\xi_{d}\left(\mathrm{nm}\right)$	10.9 ± 0.5	17.3 ± 0.4	5.6 ± 0.1	2.3 ± 0.3	2.78 ± 0.17	n/a
$R\left(\mathrm{nm}\right)$	1.29 ± 0.03	0.76 ± 0.02	1.43 ± 0.02	1.11 ± 0.02	0.55 ± 0.01	n/a
$R_{g}\left(\mathrm{nm}\right)$	n/a	n/a	n/a	n/a	3.36 ± 0.04	3.09 ± 0.18
$\xi \left(\mathrm{nm}\right)$	n/a	n/a	n/a	n/a	0.73 ± 0.01	0.77 ± 0.02
D	n/a	n/a	n/a	n/a	2.04 ± 0.04	1.39 ± 0.03

**Table 3:** Optical absorption and photoluminescent properties of poly(pyridinium salt)-fluorenes, **I**, **II**, **III**, and **V** in methanol.

Polymer	I	II ^a^	III	IV	V
λ_abs_ wavelength (nm)	340	338	340	-	340
Molar absorptivity (M^−1^ cm^−1^)	60,244 ± 2782	62,437 ± 4120	60,113 ± 2857	-	60,232 ± 2881
λ_em_ wavelength (nm)	535	534	541	-	541
AQY (Φ_F_)	0.023	0.027	0.025	-	0.022
AQY_solid_ (Φ_F_)	0.020	<0.01	0.020	-	0.034

^a^ Measured in acetonitrile.

## Data Availability

The original contributions presented in this study are included in the article. Further inquiries can be directed to the corresponding author.

## Supplementary Materials

The following supporting information can be downloaded at https://www.mdpi.com/article/10.3390/polym17131785/s1: Figure S1: FTIR spectrum of polymer **I** taken at room temperature; Figure S2: FTIR spectrum of polymer **II** taken at room temperature; Figure S3: FTIR spectrum of polymer **III** taken at room temperature; Figure S4: FTIR spectrum of polymer **IV** taken at room temperature; Figure S5: FTIR spectrum of polymer **V** taken at room temperature; Figure S6: The ^1^H and ^13^C NMR spectra of polymer **I** in *d*_6_-DMSO taken at room temperature; Figure S7: The ^1^H, ^13^C, and ^19^F NMR spectra of polymer **II** in *d*_6_-DMSO taken at room temperature; Figure S8: The ^1^H and ^13^C NMR spectra of polymer **III** in CD_3_OD taken at room temperature; Figure S9: The ^1^H and ^13^C NMR spectra of polymer **IV** in *d*_6_-DMSO taken at room temperature; Figure S10: The ^1^H and ^13^C NMR spectra of polymer **V** in CD_3_OD taken at room temperature; Figure S11: UV-Visible spectrum of polymer **I** in CH_3_OH at 1–5 × 10^−5^ M concentrations; Figure S12: UV-Visible spectrum of polymer **II** in CH_3_CN at 1–5 × 10^−5^ M concentrations; Figure S13: UV-Visible spectrum of polymer **III** in CH_3_OH at 1–5 × 10^−5^ M concentrations; Figure S14: UV-Visible spectrum of polymer **V** in CH_3_OH at 1–5 × 10^−5^ M concentrations; Figure S15: Emission/excitation spectra of polymer **I** in CH_3_OH at 5 × 10^−5^ M; Figure S16: Emission/excitation spectra of polymer **II** in CH_3_CN at 5 × 10^−5^ M; Figure S17: Emission/excitation spectra of polymer **III** in CH_3_OH at 5 × 10^−5^ M; Figure S18: Emission/excitation spectra of polymer **V** in CH_3_OH at 5 × 10^−5^ M; Figure S19: Fluorescence intensity and emission peak of polymer **I** as a function of water content in CH_3_OH (1 μM repeating units, λ_ex_ at 390 nm); Figure S20: Fluorescence intensity and emission peak of polymer **II** as a function of water content in CH_3_CN (1 μM repeating units, λ_ex_ at 390 nm); Figure S21: Fluorescence intensity and emission peak of polymer **III** as a function of water content in CH_3_OH (1 μM repeating units, λ_ex_ at 390 nm); Figure S22: Fluorescence intensity and emission peak of polymer **III** as a function of water content in CH_3_CN (1 μM repeating units, λ_ex_ at 390 nm); Figure S23: Fluorescence intensity and emission peak of polymer **V** as a function of water content in CH_3_OH (1 μM repeating units, λ_ex_ at 390 nm); Figure S24: Fluorescence intensity and emission peak of polymer **V** as a function of water content in CH_3_CN (1 μM repeating units, λ_ex_ at 390 nm); Figure S25: Emission spectra of polymer **I** (1 μM repeating units, excited at 390 nm) in DMSO/H_2_O mixtures with varying amounts water % (*v*/*v*); Figure S26: Emission spectra of polymer **I** (1 μM repeating units, excited at 390 nm) in CH_3_OH/H_2_O mixtures with varying amounts water % (*v*/*v*); Figure S27: Emission spectra of polymer **II** (1 μM repeating units, excited at 390 nm) in DMSO/H_2_O mixtures with varying amounts of water % (*v*/*v*); Figure S28: Emission spectra of polymer **II** (1 μM repeating units, excited at 390 nm) in CH_3_CN/H_2_O mixtures with varying amounts of water % (*v*/*v*); Figure S29: Emission spectra of polymer **III** (1 μM repeating units, excited at 390 nm) in DMSO/H_2_O mixtures with varying amounts of water % (*v*/*v*); Figure S30: Emission spectra of polymer **III** (1 μM repeating units, excited at 390 nm) in CH_3_OH/H_2_O mixtures with varying amounts of water % (*v*/*v*); Figure S31: Emission spectra of polymer **III** (1 μM repeating units, excited at 390 nm) in CH_3_CN/H_2_O mixtures with varying amounts of water % (*v*/*v*); Figure S32: Emission spectra of polymer **V** (1 μM repeating units, excited at 390 nm) in DMSO/H_2_O mixtures with varying amounts of water % (*v*/*v*); Figure S33: Emission spectra of polymer **V** (1 μM repeating units, excited at 390 nm) in CH_3_OH/H_2_O mixtures with varying amounts of water % (*v*/*v*); Figure S34: Emission spectra of polymer **V** (1 μM repeating units, excited at 390 nm) in CH_3_CN/H_2_O mixtures with varying amounts of water % (*v*/*v*).
