# Supplementary material for: Poly(pyridinium salt)s Containing 9,9-Bis(4-aminophenyl)fluorene Moieties with Various Organic Counterions Exhibiting Both Lyotropic Liquid-Crystalline and Light-Emitting Properties
Source: Polymers (Basel). 2025 Jun 27;17(13):1785. doi: 10.3390/polym17131785 (PMC12252291; doi:10.3390/polym17131785)
Supplement: Supplementary file 1 [file polymers-17-01785-s001.zip › polymers-3657006-supplementary.pdf]

Supplementary Information

Poly(pyridinium salt)s Containing 9,9-Bis(4-Aminophenyl)fluorene Moieties with Various Organic Counterions Exhibiting Both Lyotropic Liquid-Crystalline and Light-Emitting Properties

Pradip K. Bhowmik[1*], David King[1], Haesook Han[1], András F. Wacha[2], and Matti Knaapila[3]

[1]Department of Chemistry and Biochemistry, University of Nevada Las Vegas, 4505 Maryland Parkway, Box 454003, Las Vegas, Nevada 89154, USA
[2]Research Center for Natural Sciences, Institute of Materials and Environmental Chemistry, Magyar Tedesko köútja 2, H-1117 Budapest, Hungary
[3]Department of Physics, Norwegian University of Science and Technology, Høgskoleringen 5 7491 Trondheim, Norway

*Correspondence:
Pradip K. Bhowmik: pradip.bhowmik@unlv.edu

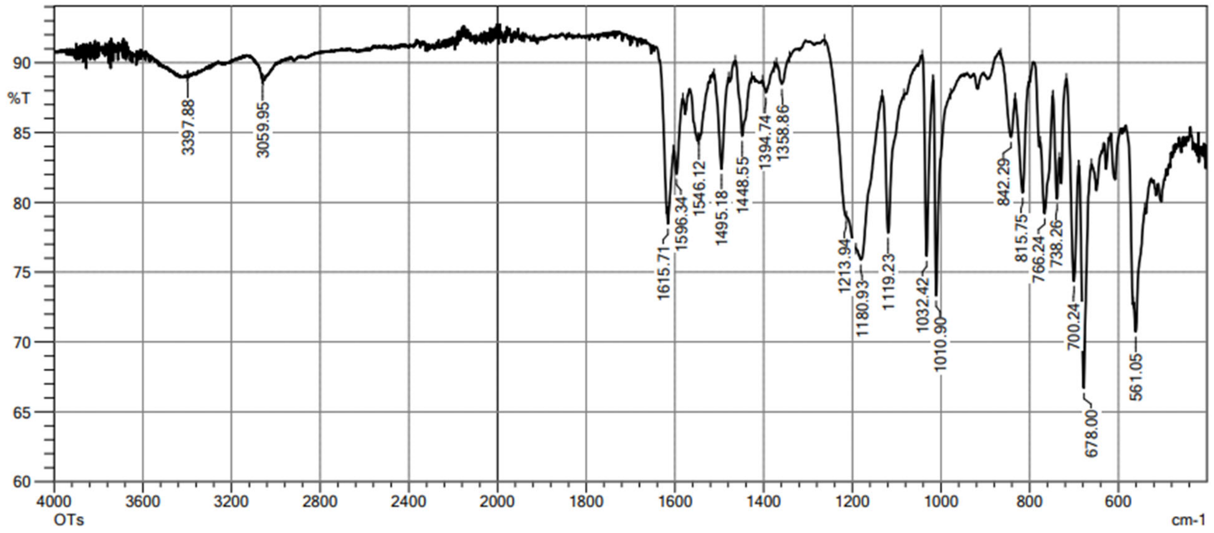

Figure S1. FTIR spectrum of polymer **I** taken at room temperature.

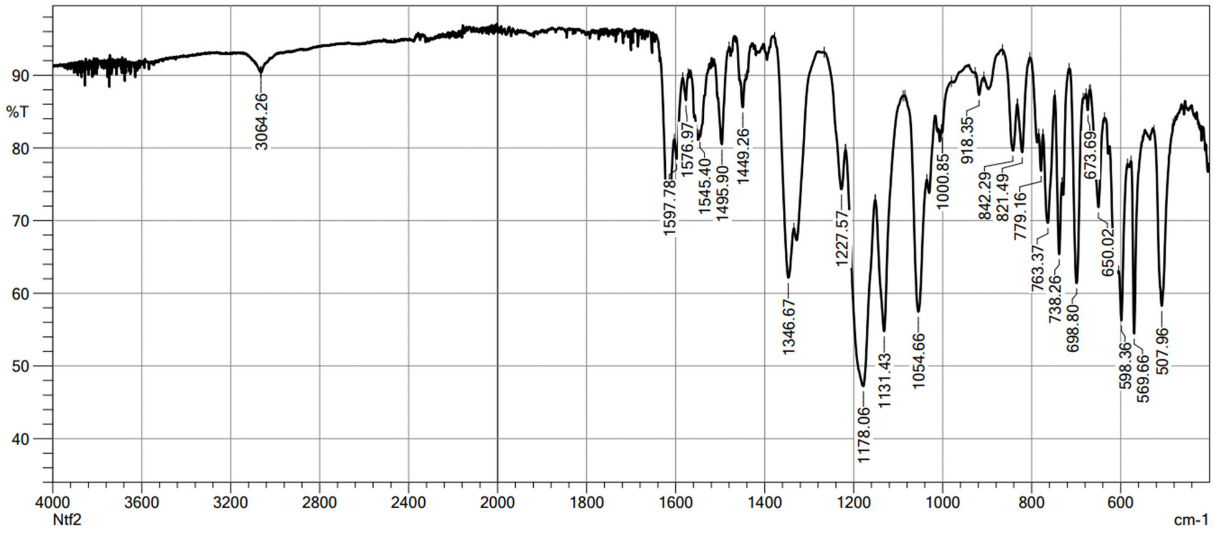

Figure S2. FTIR spectrum of polymer **II** taken at room temperature.

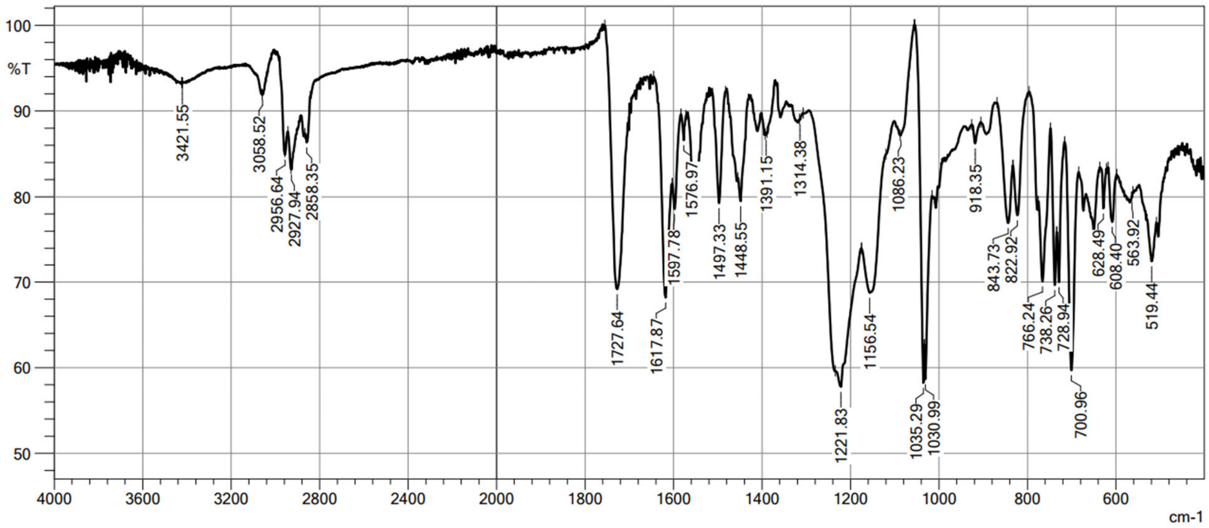

Figure S3. FTIR spectrum of polymer **III** taken at room temperature.

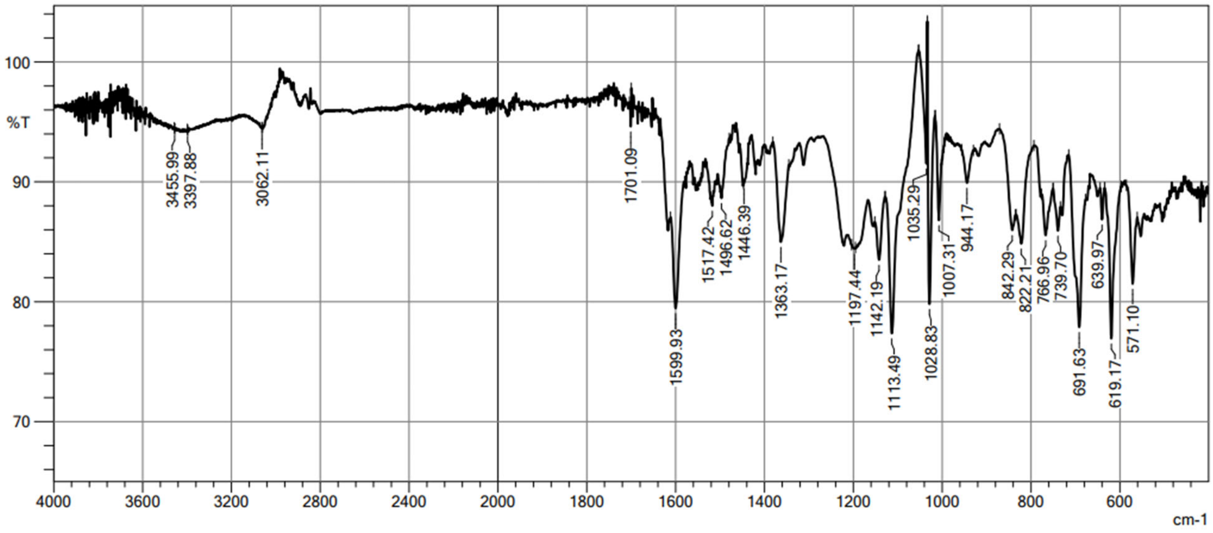

Figure S4. FTIR spectrum of polymer **IV** taken at room temperature.

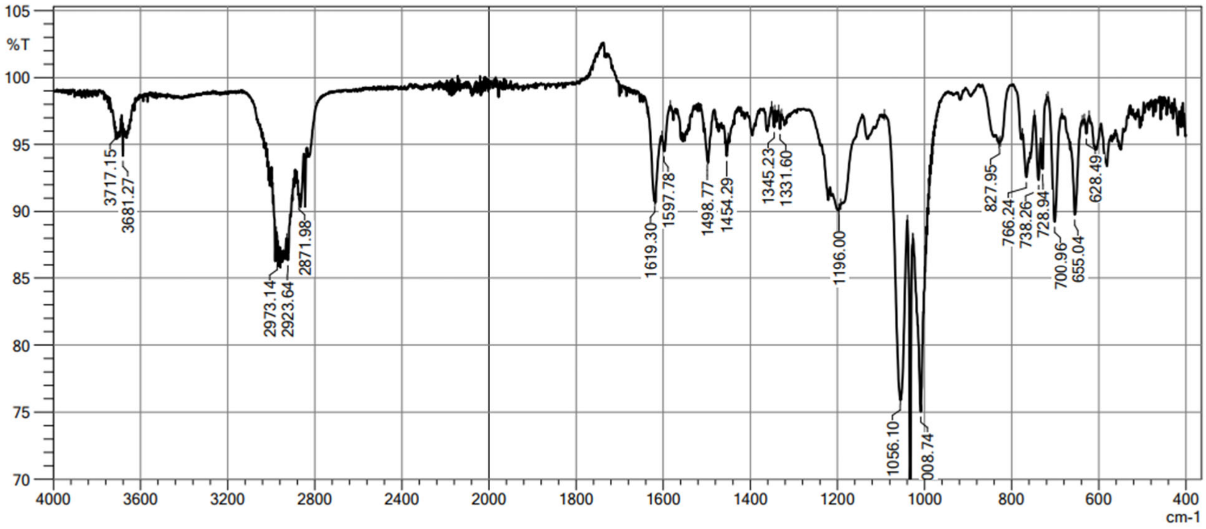

Figure S5. FTIR spectrum of polymer **V** taken at room temperature.

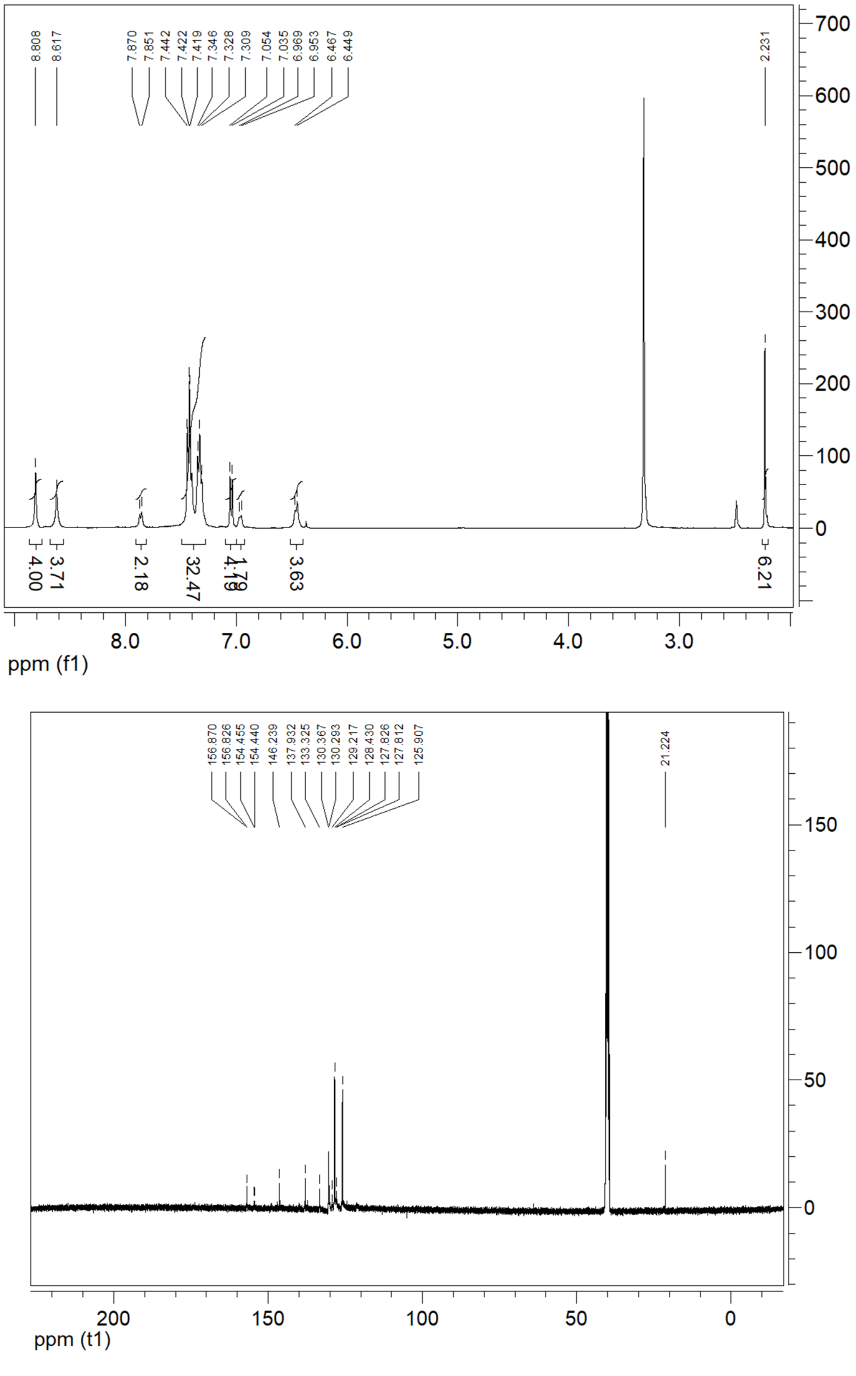

Figure S6. ^{1}H and ^{13}C NMR spectra of polymer **I** in d6-DMSO taken at room temperature.

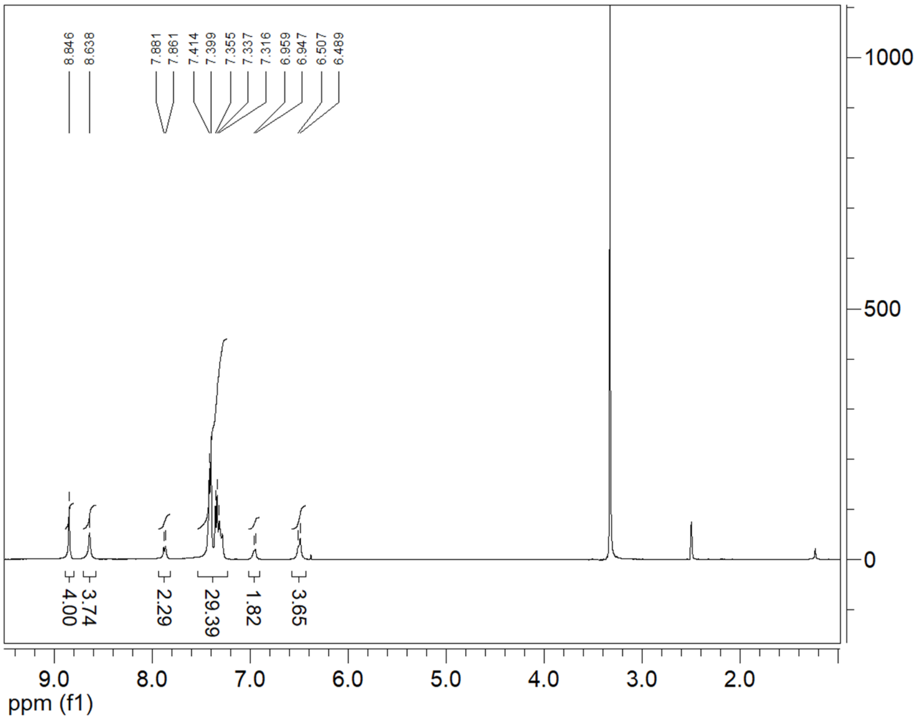
8.846
8.638
7.881
7.861
7.414
7.399
7.355
7.337
7.316
6.959
6.947
6.507
6.489
4.00
3.74
2.29
29.39
1.82
3.65
1000
500
0
9.0
8.0
7.0
6.0
5.0
4.0
3.0
2.0
ppm (f1)

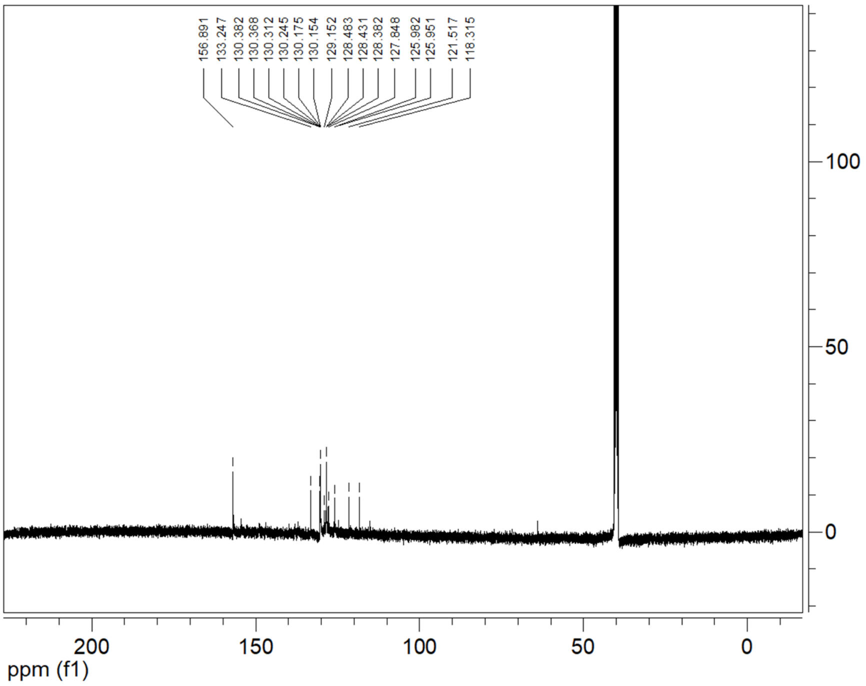
156.891
133.247
130.382
130.368
130.312
130.245
130.175
130.154
129.152
128.483
128.431
128.382
127.848
125.982
125.951
121.517
118.315
100
50
0
200
150
100
50
0
ppm (f1)

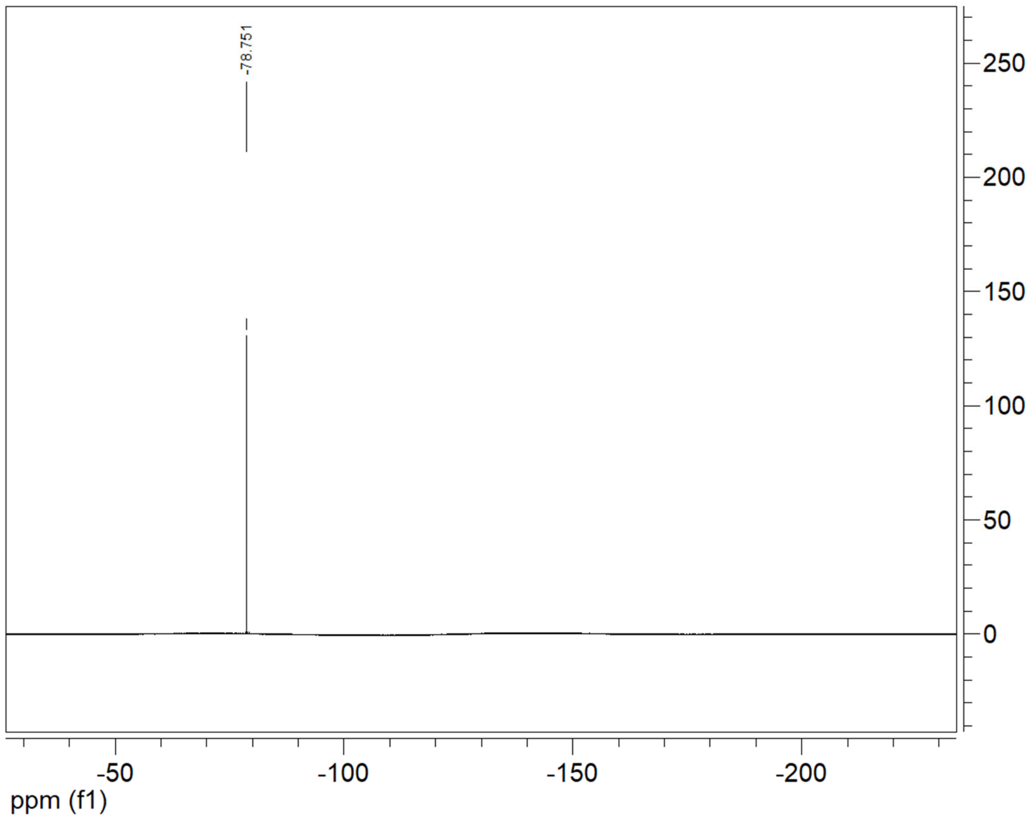

Figure S7. ^{1}H, ^{13}C and ^{19}F NMR spectra of polymer **II** in d_6-DMSO taken at room temperature.

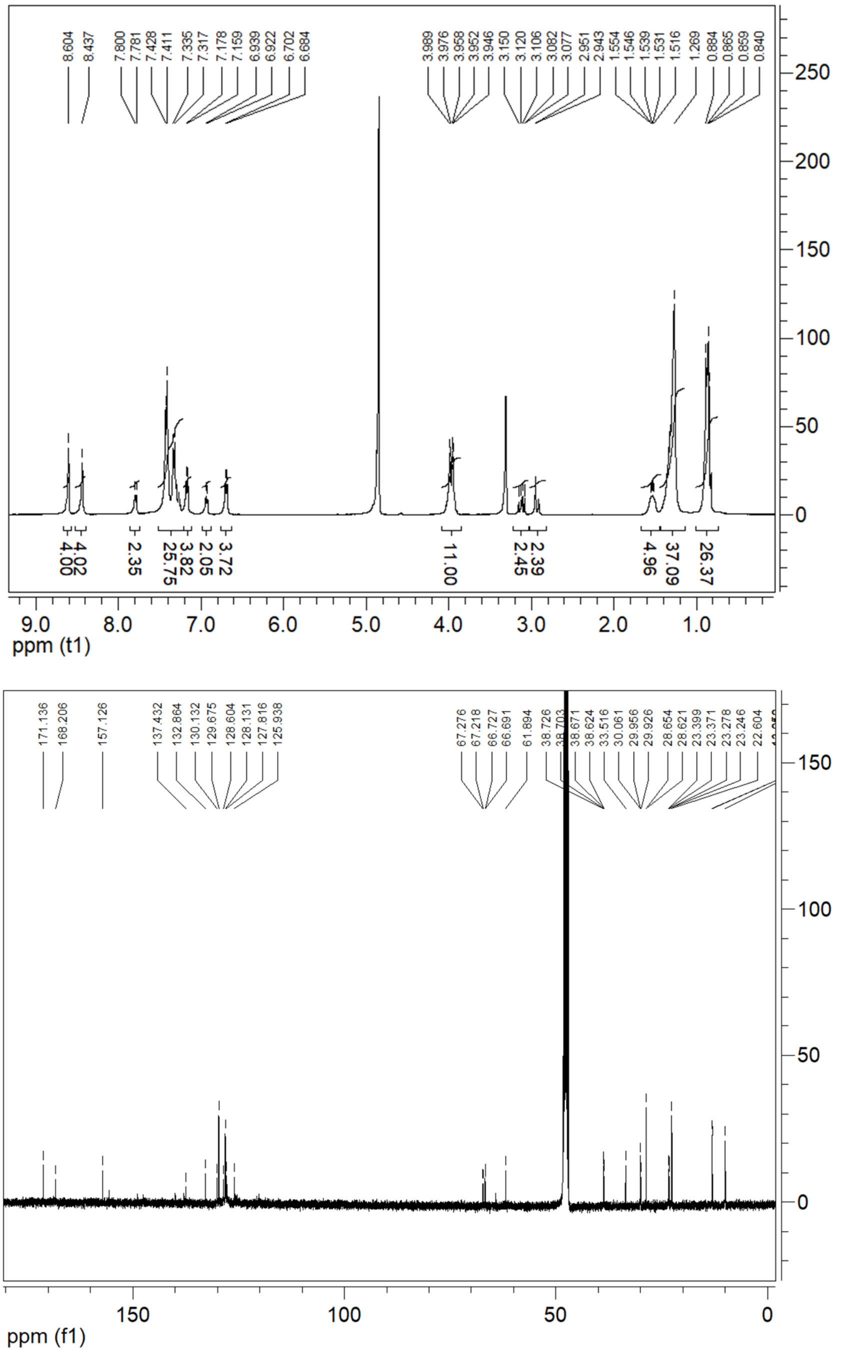

Figure S8. ^{1}H and ^{13}C NMR spectra of polymer **III** in CD_3OD taken at room temperature.

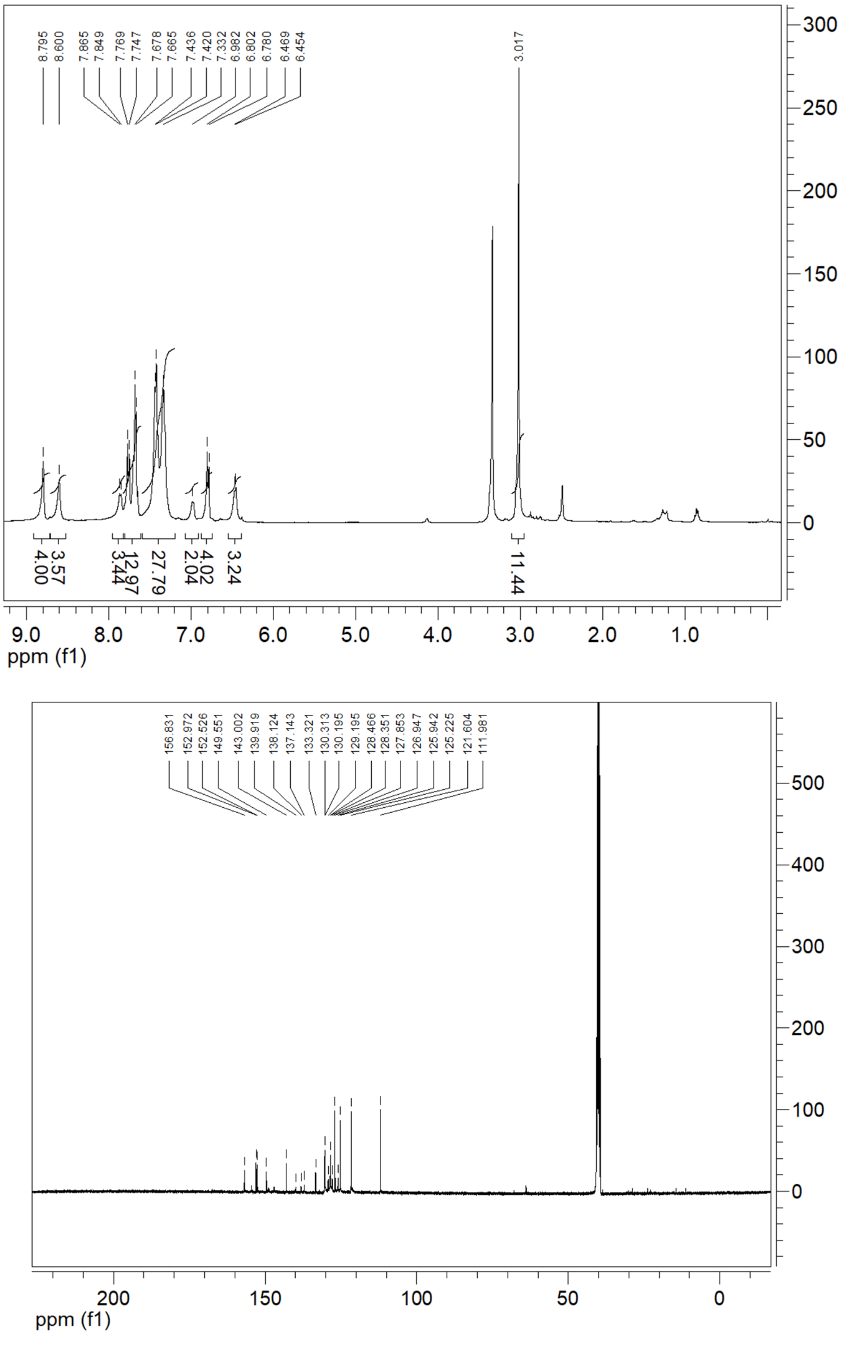

Figure S9. ^{1}H and ^{13}C NMR spectra of polymer **IV** in d_6-DMSO taken at room temperature.

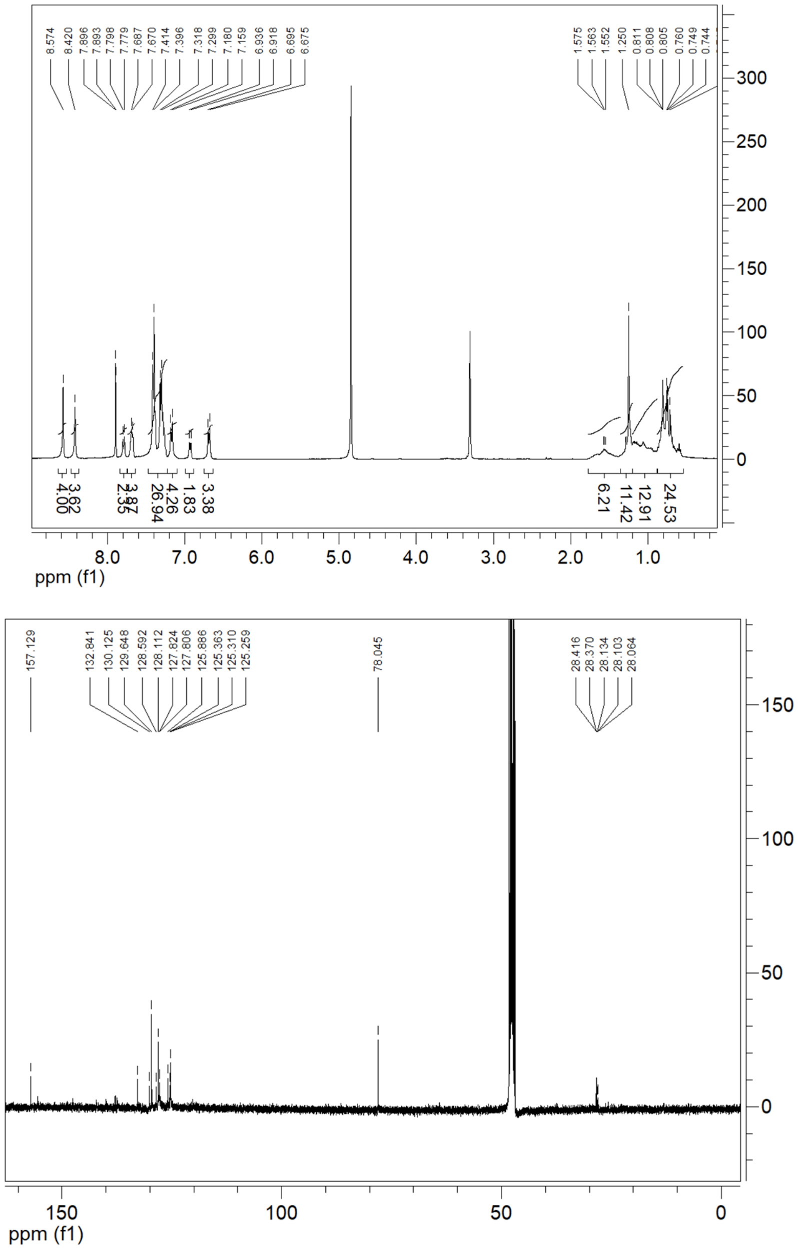

Figure S10. ^{1}H and ^{13}C NMR spectra of polymer **V** in CD_3OD taken at room temperature.

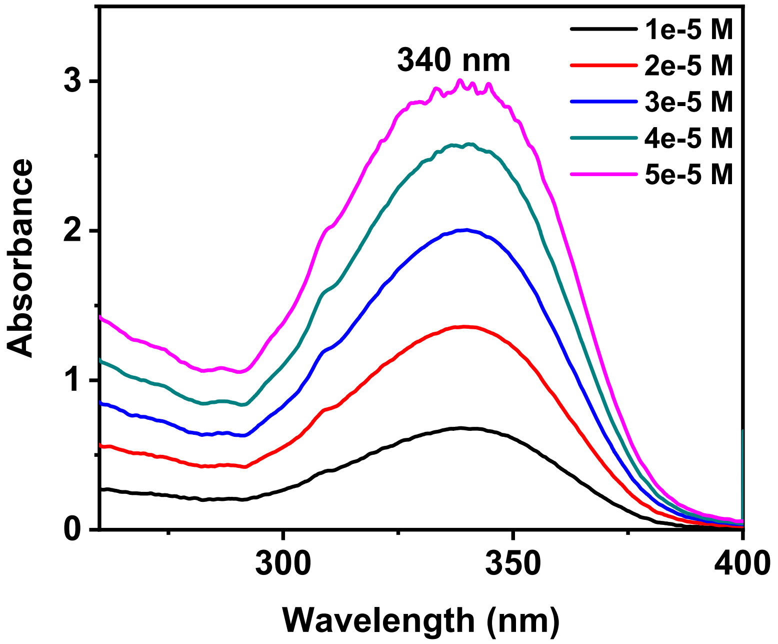

Figure S11. UV-Visible spectrum of polymer **I** in CH_3OH at 1-5 x 10^{-5} M concentrations.

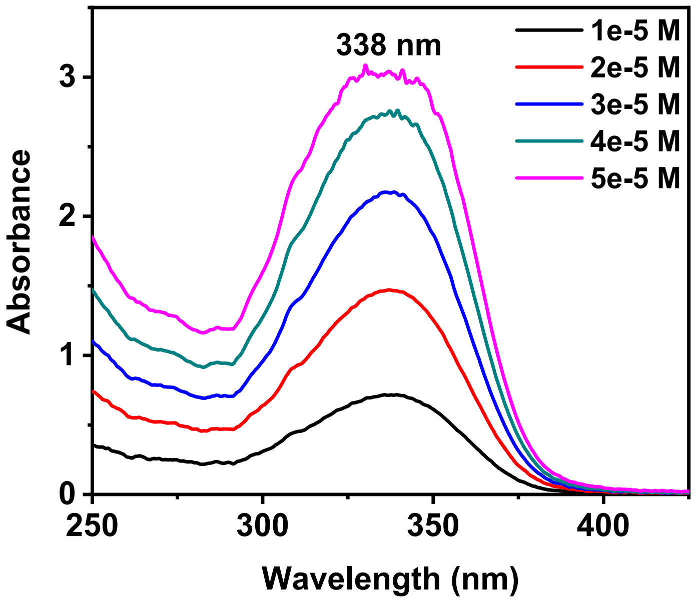

Figure S12. UV-Visible spectrum of polymer **II** in CH_3CN at 1-5 x 10^{-5} M concentrations.

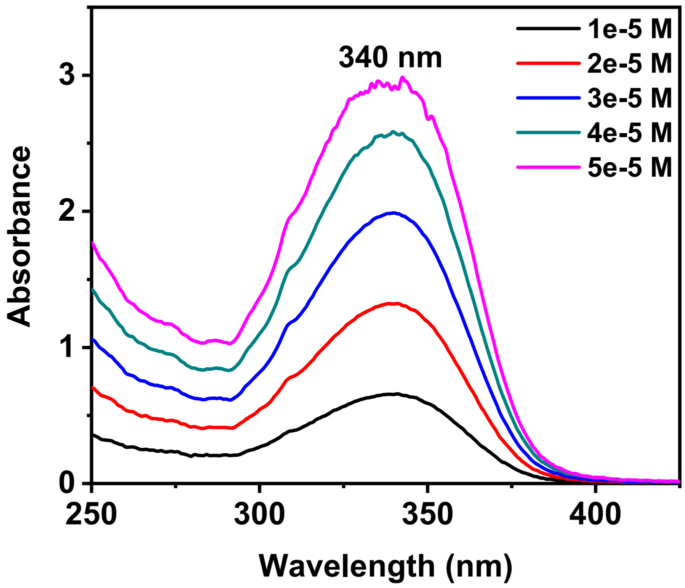

Figure S13. UV-Visible spectrum of polymer **III** in CH_3OH at 1-5 x 10^{-5} M concentrations.

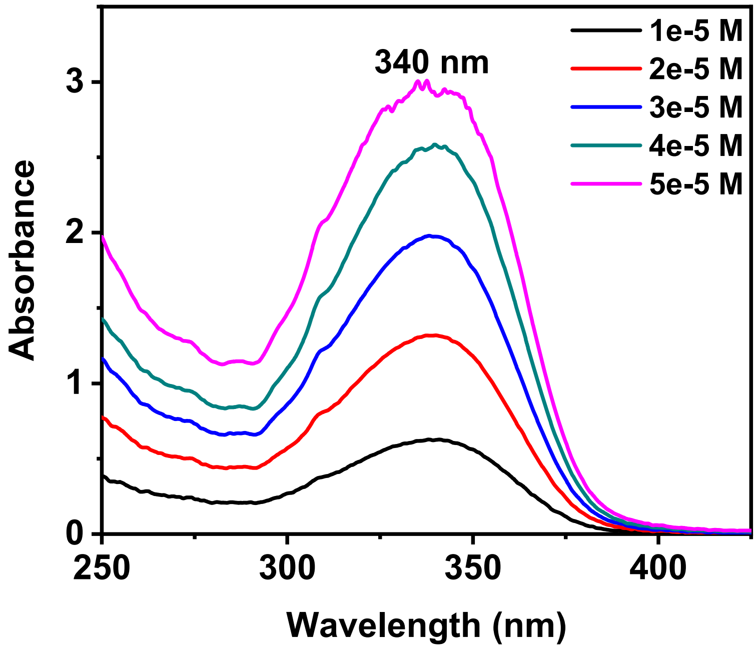

Figure S14. UV-Visible spectrum of polymer **V** in CH_3OH at 1-5 x 10^{-5} M concentrations.

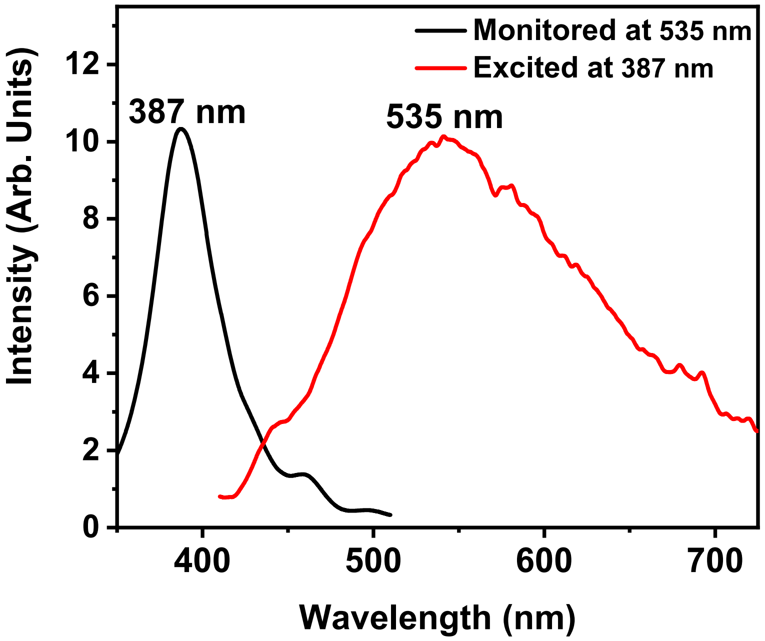

Figure S15. Emission/excitation spectra of polymer **I** in CH_3OH at 5 x 10^{-5} M.

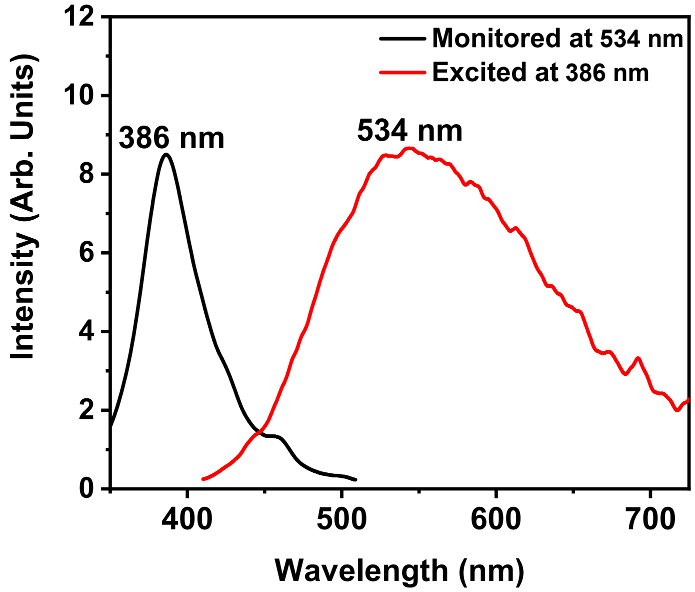

Figure S16. Emission/excitation spectra of polymer **II** in CH_3CN at 5 x 10^{-5} M.

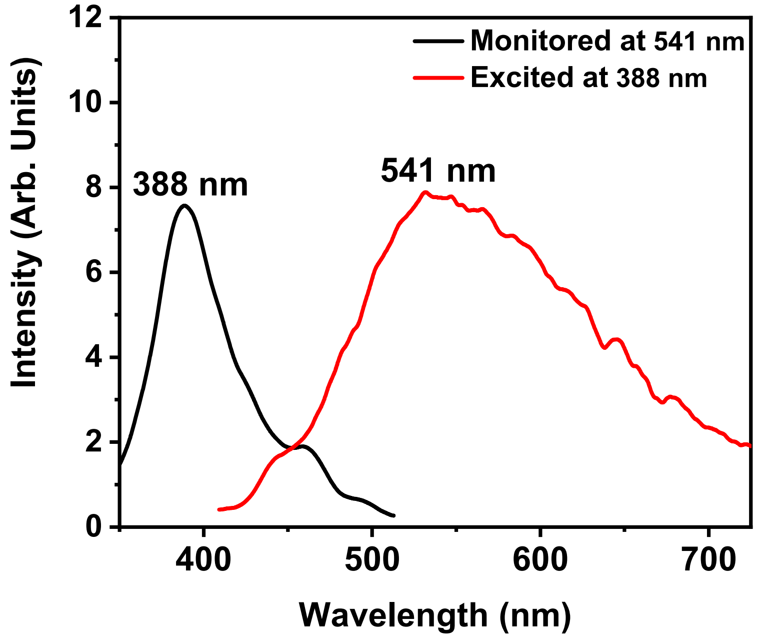

Figure S17. Emission/excitation spectra of polymer **III** in CH_3OH at 5 x 10^{-5} M.

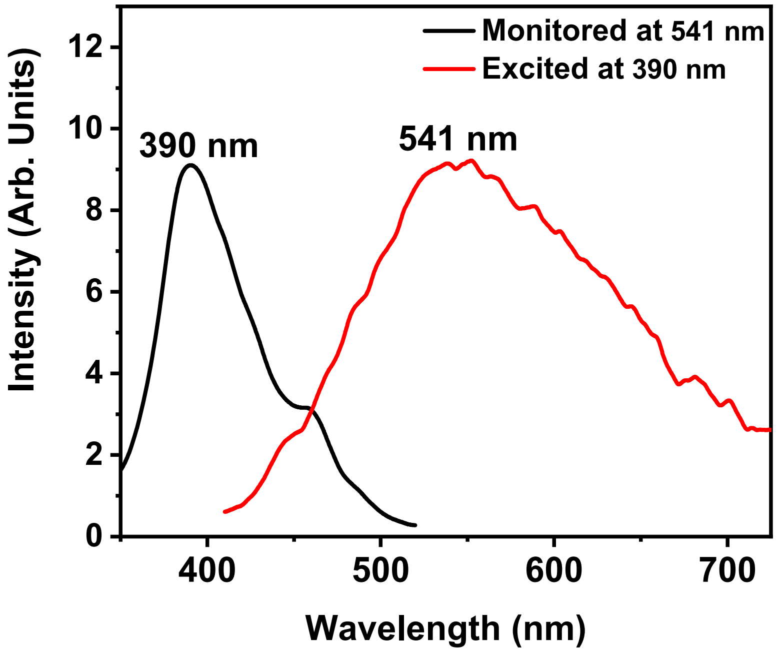

Figure S18. Emission/excitation spectra of polymer **V** in CH_3OH at 5 x 10^{-5} M.

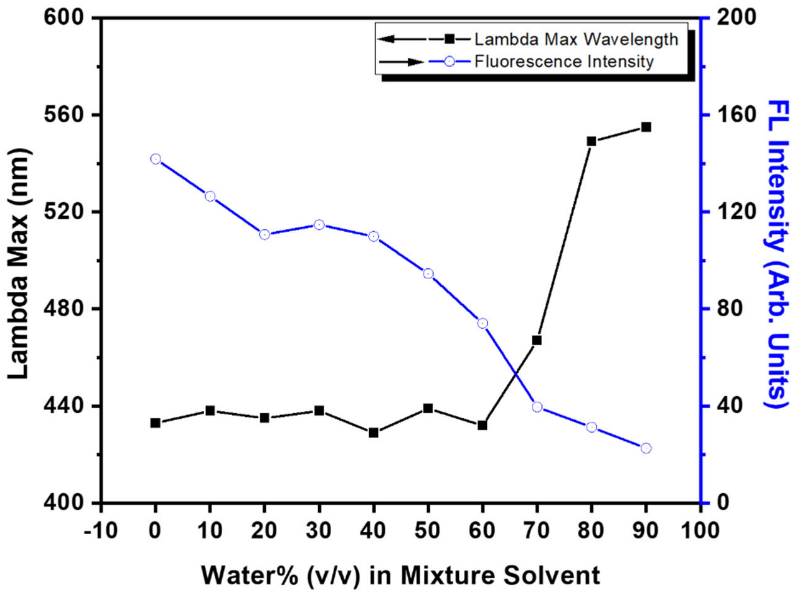

Figure S19. Fluorescence intensity and emission peak of polymer **I** as a function of water content in CH_3OH (1 μM repeating units, λ_{ex} at 390 nm).

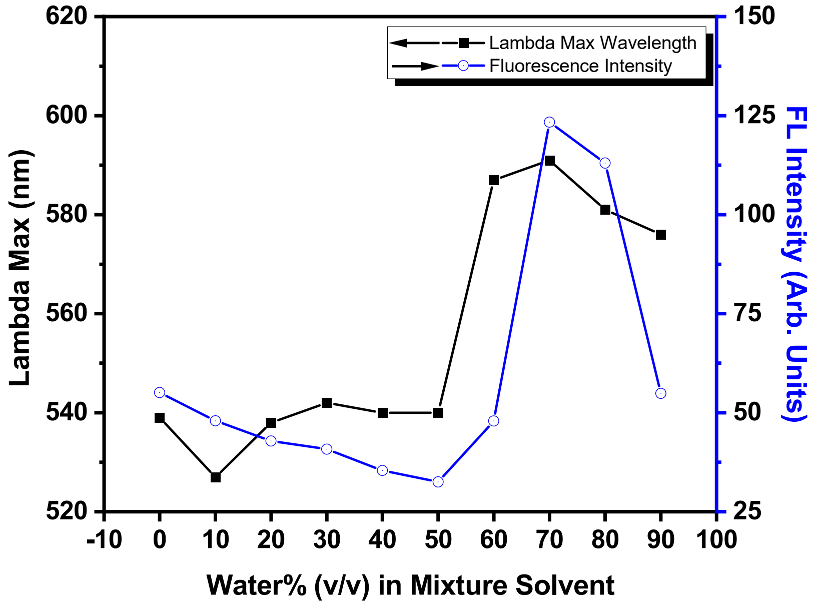

Figure S20. Fluorescence intensity and emission peak of polymer **II** as a function of water content in CH_3CN (1 μM repeating units, λ_{ex} at 390 nm).

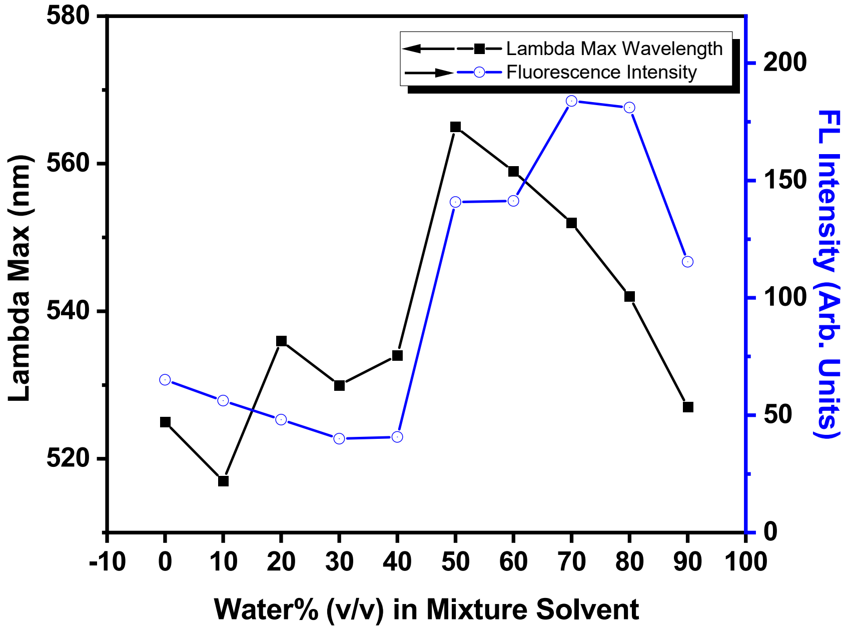

Figure S21. Fluorescence intensity and emission peak of polymer **III** as a function of water content in CH_3OH (1 μM repeating units, λ_{ex} at 390 nm).

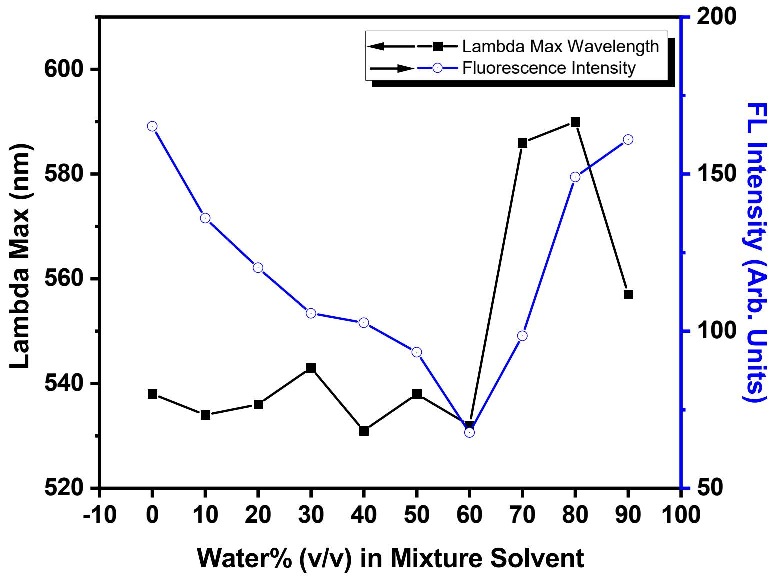

Figure S22. Fluorescence intensity and emission peak of polymer **III** as a function of water content in CH_3CN (1 μM repeating units, λ_{ex} at 390 nm).

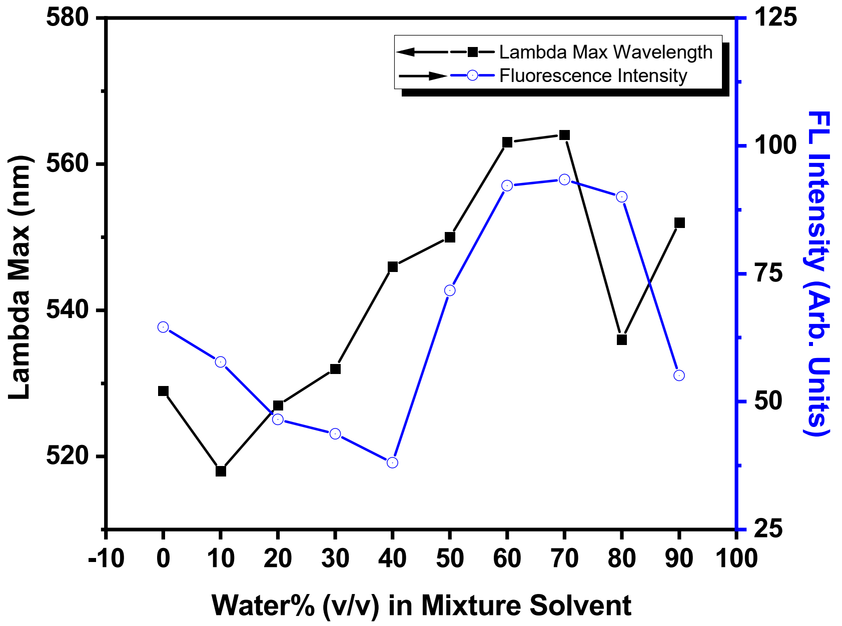

Figure S23. Fluorescence intensity and emission peak of polymer **V** as a function of water content in CH_3OH (1 μM repeating units, λ_{ex} at 390 nm).

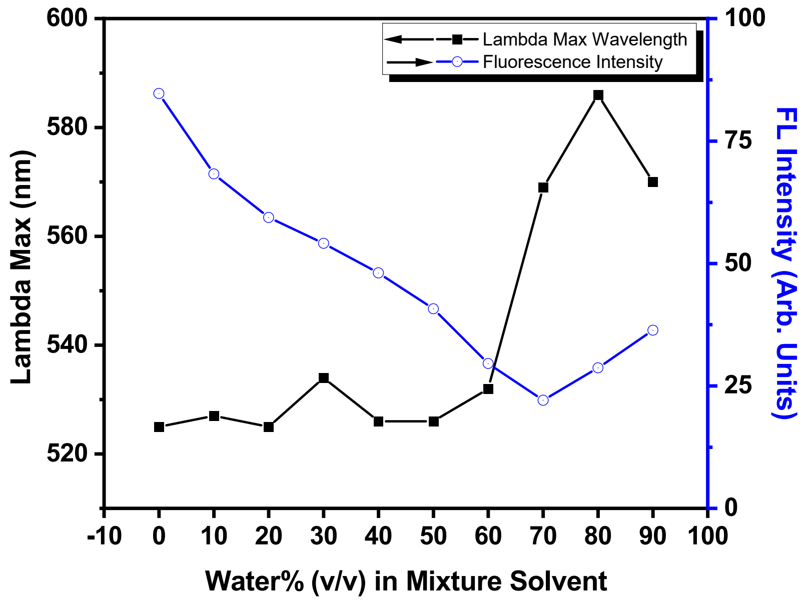

Figure S24. Fluorescence intensity and emission peak of polymer **V** as a function of water content in CH_3CN (1 μM repeating units, λ_{ex} at 390 nm).

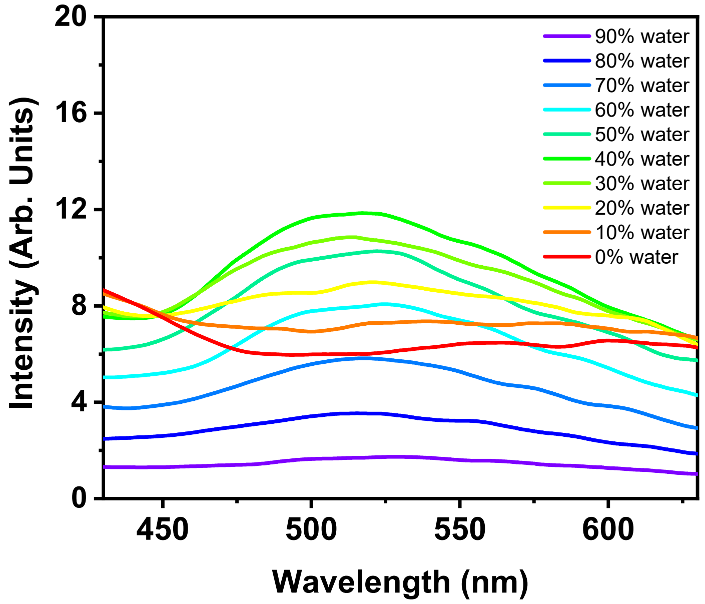

Figure S25. Emission spectra of polymer **I** (1 μM repeating units, excited at 390 nm) in DMSO/H_2O mixtures with varying amounts water % (v/v).

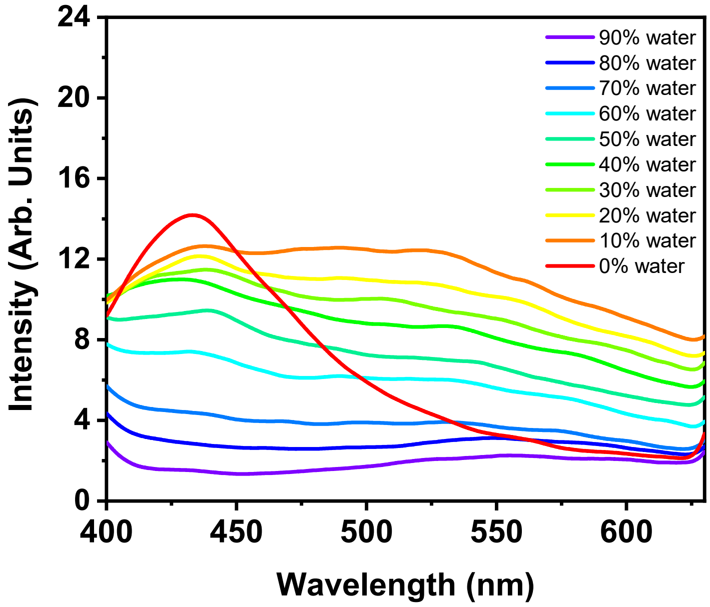

Figure S26. Emission spectra of polymer **I** (1 µM repeating units, excited at 390 nm) in CH_3OH/H_2O mixtures with varying amounts water % (v/v).

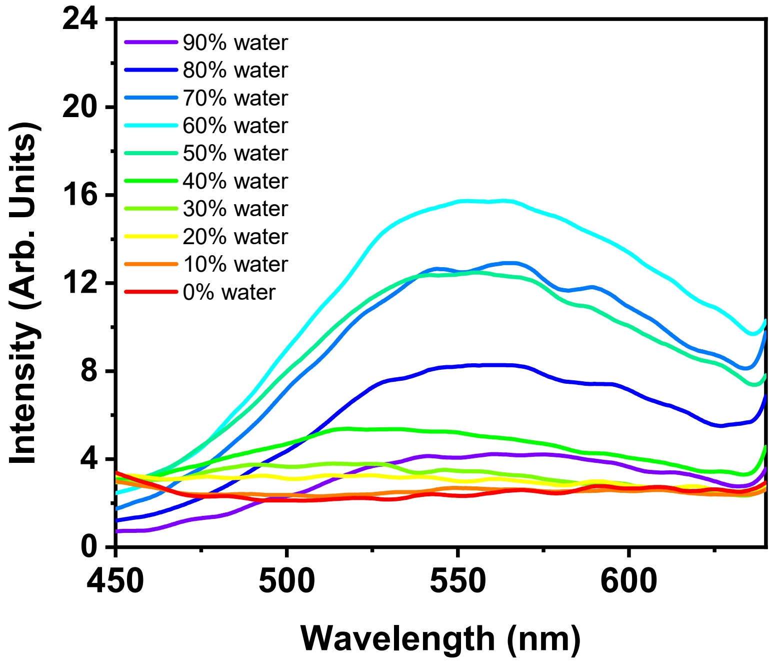

Figure S27. Emission spectra of polymer **II** (1 µM repeating units, excited at 390 nm) in DMSO/H_2O mixtures with varying amounts water % (v/v).

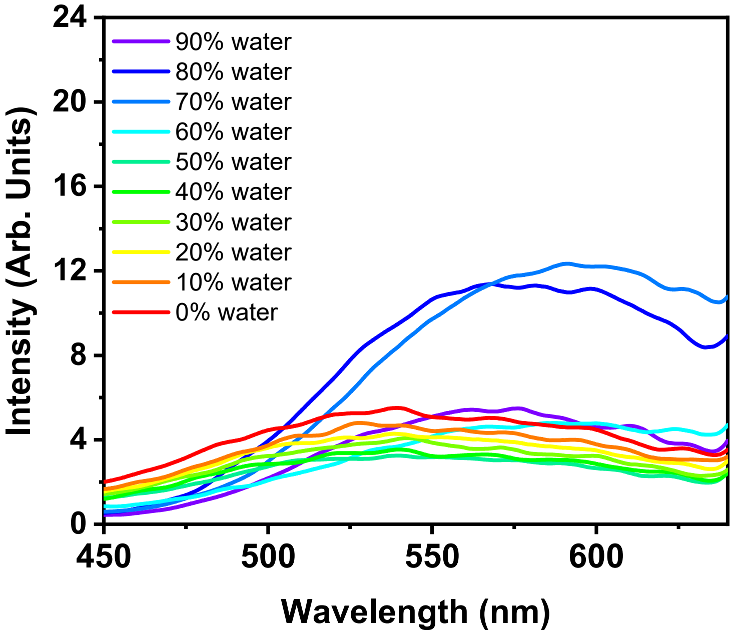

Figure S28. Emission spectra of polymer **II** (1 μM repeating units, excited at 390 nm) in CH_3CN/H_2O mixtures with varying amounts water % (v/v).

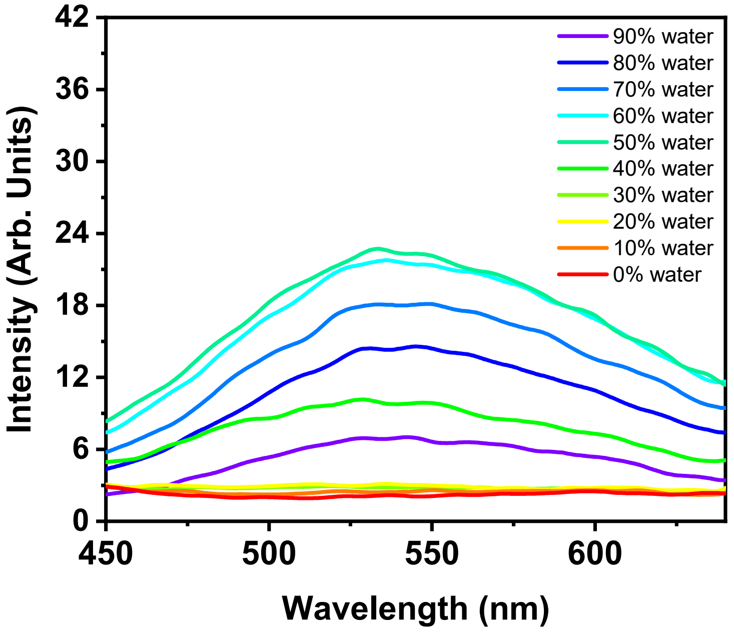

Figure S29. Emission spectra of polymer **III** (1 μM repeating units, excited at 390 nm) in DMSO/H_2O mixtures with varying amounts water % (v/v).

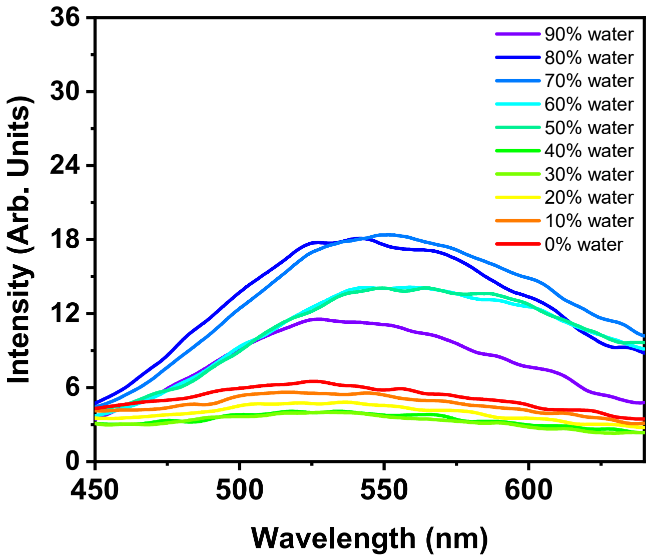

Figure S30. Emission spectra of polymer **III** (1 μM repeating units, excited at 390 nm) in CH_3OH/H_2O mixtures with varying amounts water % (v/v).

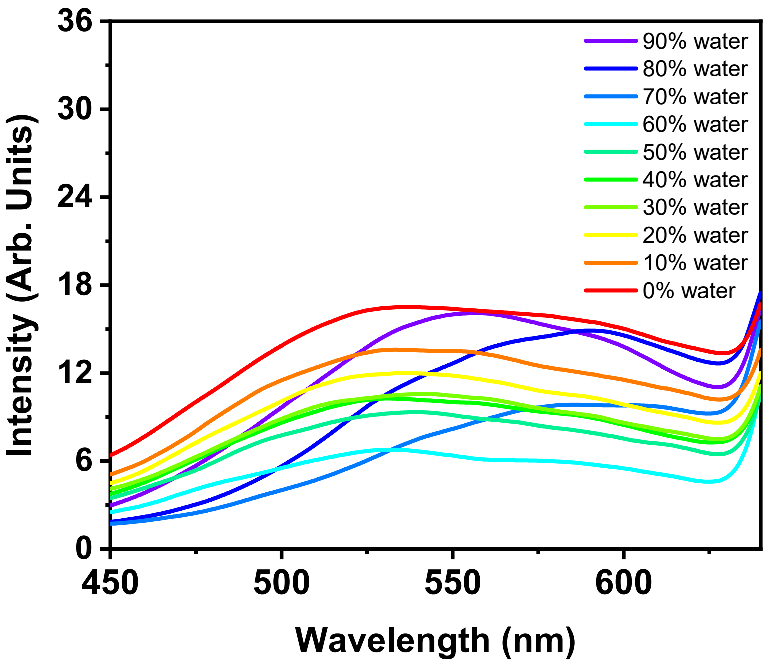

Figure S31. Emission spectra of polymer **III** (1 µM repeating units, excited at 390 nm) in CH_3CN/H_2O mixtures with varying amounts water % (v/v).

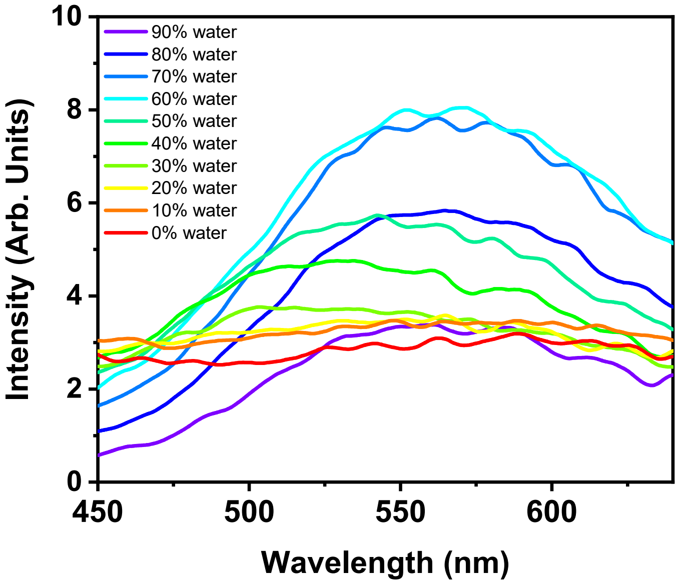

Figure S32. Emission spectra of polymer **V** (1 µM repeating units, excited at 390 nm) in $DMSO/H_2O$ mixtures with varying amounts water % (v/v).

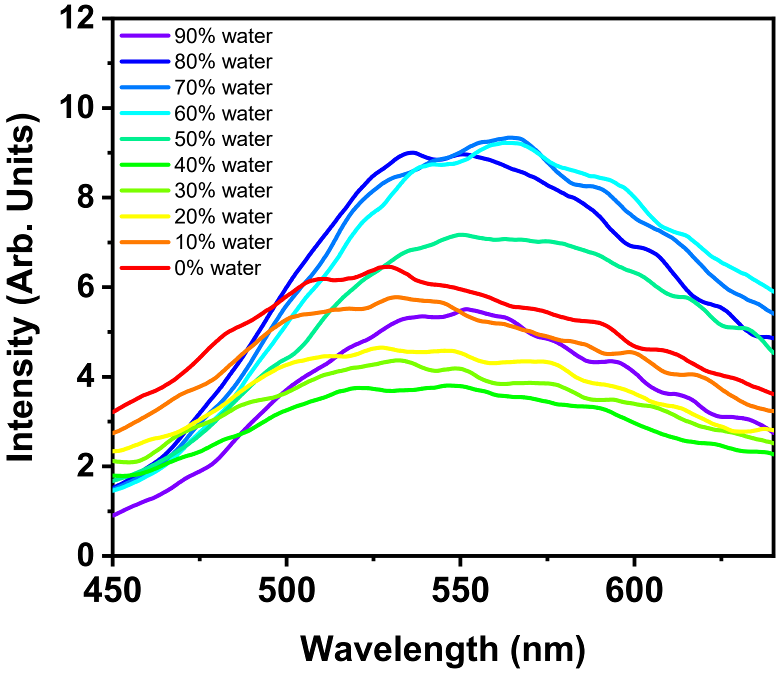

Figure S33. Emission spectra of polymer **V** (1 µM repeating units, excited at 390 nm) in CH_3OH/H_2O mixtures with varying amounts water % (v/v).

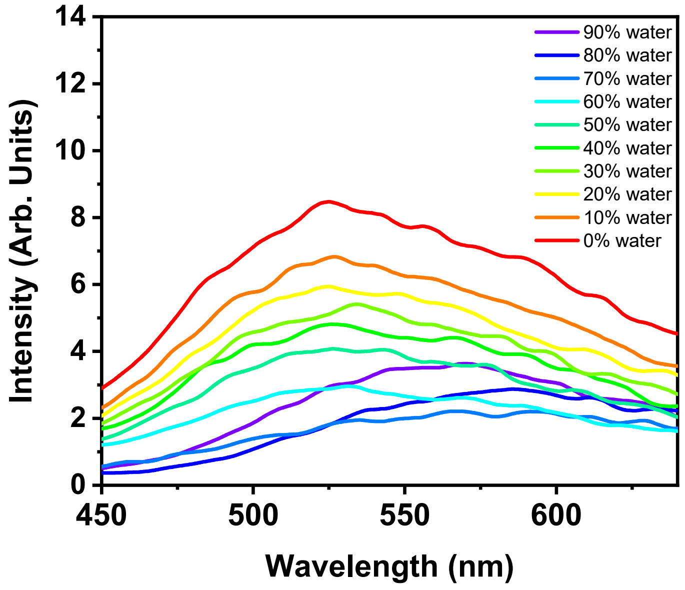

Figure S34. Emission spectra of polymer **V** (1 µM repeating units, excited at 390 nm) in CH_3CN/H_2O mixtures with varying amounts water % (v/v).
